# Polymerization and flanking domains of the bactofilin BacA collectively regulate stalk formation in *Asticcacaulis biprosthecum*

**DOI:** 10.1371/journal.pgen.1011542

**Published:** 2025-08-13

**Authors:** Maxime Jacq, Paul D. Caccamo, Yves V. Brun

**Affiliations:** 1 Département de microbiologie, infectiologie et immunologie, Université de Montréal, Montréal, Québec, Canada; 2 Biodesign Center for Mechanisms of Evolution and School of Life Sciences, Arizona State University, Tempe, Arizona, United States of America; 3 Howard Hughes Medical Institute, Chevy Chase, Maryland, United States of America; 4 Department of Biology, Indiana University, Bloomington, Indiana, United States of America; Michigan State University, UNITED STATES OF AMERICA

## Abstract

Bactofilins are a recently discovered class of cytoskeletal protein, widely implicated in subcellular organization and morphogenesis in bacteria and archaea. Several lines of evidence suggest that bactofilins polymerize into filaments using a central β-helical core domain, flanked by variable N- and C- terminal domains that may be important for scaffolding and other functions. In *Asticcacaulis biprosthecum*, the bactofilin BacA serves as a topological organizer of stalk synthesis, localizing to the stalk base and coordinating the synthesis of these long, thin extensions of the cell envelope. The easily distinguishable phenotypes of wild-type *A. biprosthecum* stalks and *Δ*bacA** “pseudostalks” make this an ideal system for investigating how mutations in BacA affect its functions in morphogenesis. Here, we redefine the core domain of *A. biprosthecum* BacA using various bioinformatics and biochemical approaches to precisely delimit the N- and C- terminal domains. We then show that loss of these terminal domains leads to cells with severe morphological abnormalities, typically presenting a pseudostalk phenotype. BacA mutants lacking the N- and C- terminal domains also exhibit localization defects, implying that the terminal domains of BacA may be involved in its subcellular positioning, possibly through regulatory interactions with membrane-associated factors or other morphological proteins. We further show that point mutations that render BacA defective for polymerization lead to stalk synthesis defects. Overall, our study suggests that BacA’s polymerization capacity and domain-mediated cellular positioning play a crucial role in the protein’s function as a morphogenic regulator.

## Author summary

Bacteria exhibit a wide variety of shapes and structures, many of which are crucial for their cellular functions. Among these structures is the stalk—a thin, tubular extension of the cell envelope formed by bacteria such as *Asticcacaulis biprosthecum*. Stalk synthesis in *Asticcacaulis biprosthecum* relies on the bactofilin BacA, a self-polymerizing cytoskeletal protein, whose deletion results in the dysregulation of stalk synthesis and the formation of short, stubby “pseudostalks”. We use this unique phenotype to characterize the subdomains of BacA, and find that BacA’s ability to coordinate stalk synthesis depends on its conserved polymerization domain as well as its flanking N- and C-terminal domains, which are essential for proper localization. Our findings highlight how bactofilins combine conserved and variable regions to generate complex structures that serve as a platform for evolving new functions.

## Introduction

All cells require spatiotemporal organization of their components and processes for proper function. While bacteria lack the typical membrane-bound organelles that allow for higher-order partitioning in eukaryotic systems, they possess cytoskeletal scaffolding proteins for spatiotemporal organization of protein complexes. Bacterial cytoskeletal scaffolds facilitate the localization and interplay of proteins and their regulatory elements at specific subcellular locations at specific times, partitioning the cytoplasm into functionally distinct domains. Given this role, bacterial cytoskeletal scaffolds serve as key regulators of many important processes in bacterial cells, including cell growth, cell division, DNA segregation, establishment of cell polarity, morphology, and magnetotaxis [1,2].

The most studied bacterial cytoskeletal scaffolds are MreB [3], FtsZ [4], and Crescentin [5], which represent large families of prokaryotic homologs of actin, tubulin, and intermediate filaments, respectively. However, bacteria also possess more diverse cytoskeletal scaffolds [6]. Among these are the bactofilins, a recently discovered class of cytoskeletal proteins that are widely distributed in bacteria and archaea, and occasionally found in a few eukaryotes [7–11]. Bactofilin proteins are characterized by a core bactofilin domain (DUF583) of ~100 amino acids, flanked by variable N-and C-terminal regions [8–10,12–14]. Like other bacterial cytoskeletal proteins, bactofilins are known to polymerize. Nonetheless, little is known about how the specific domains and polymerization properties of bactofilins affect their functions *in vivo*.

Functionally, bactofilins are often implicated in cellular morphogenesis [15–18], likely through their mediating role in the synthesis of the peptidoglycan (PG) cell wall, a bacterial exoskeleton that gives cells their shape. In *Proteus mirabilis*, deletion or truncation of the bactofilin *ccmA* results in highly curved, morphologically defective cells [16]. In *Myxococcus xanthus*, the bactofilin BacM assembles into fibers throughout the cell, and deletion of *bacM* results in crooked-rod shaped cells that show increased sensitivity to antibiotics targeting the cell wall [18,19]. In *Helicobacter pylori, ccmA* deletion leads to loss of the cell’s characteristic helical shape, and truncation of the N-terminal region of CcmA results in a phenotype similar to *∆ccmA* [15,20,21]*. H. pylori* CcmA interacts with various morphological proteins such as Csd5 and Csd7, using its bactofilin domain [15,20]. Among these, Csd7 is known to be important for the stability of a PG hydrolase called Csd1, further supporting a role for bactofilins in the synthesis and remodeling of the PG cell wall [20].

Bactofilins are also involved in stalk synthesis in the Alphaproteobacteria [9,22–24]. Stalks are thin, cylindrical projections of the cell envelope, which likely function as nutrient uptake antennae in stalk-forming species of the *Caulobacter* and *Asticcacaulis* genera [25–27]. Interestingly, many of these bacteria attach to surfaces via a polar adhesin. Polar stalks thus allow cell bodies to extend away from attached surfaces, while non-polar stalks help nutrient absorption by increasing the cell’s surface area and outward reach into the surrounding environment.

Synthesis of the stalk requires a specialized form of zonal PG synthesis in a spatially constrained zone from which the stalk is extruded [28–31]. In *Caulobacter crescentus,* the bactofilins BacA and BacB contribute to the elongation of a single, polar stalk, interacting with the PG synthase PbpC at the stalked pole [9,12]. Although BacA and BacB are not essential for stalk synthesis in *C. crescentus*, their deletion leads to a reduction in stalk length*.* On the other hand, in the related stalked species *Asticcacaulis biprosthecum*, which has bilateral stalks at the midcell, the bactofilin BacA serves as an important topological organizer of stalk synthesis - it localizes at the base of each stalk, where it anchors the developmental regulator of stalk synthesis SpmX, which in turn regulates PG synthesis at the base of the stalk. In the absence of BacA, *A. biprosthecum* cells produce shorter and wider protrusions in place of the stalk. These “pseudostalks” are characterized by unregulated PG insertion, following mislocalization of SpmX throughout the pseudostalk [32]. Interestingly, in certain Alphaproteobacteria, stalks are used for reproduction, budding off daughter cells from their tips [33,34]. In one such budding bacterium, *Hyphomonas neptunium*, deletion of the bactofilin genes leads to multiple, irregularly shaped, misplaced stalks, which later produce amorphous bulges corresponding to unconstrained PG insertion during bud formation [23]. Thus, the bactofilins play an important role in regulating zonal growth for stalk synthesis, potentially through interactions with stalk-specific developmental regulators and PG synthetic complexes.

The bactofilins’ ability to polymerize appears to be important for their function. Polymerization is thought to be mediated via hydrophobic interactions between conserved hydrophobic residues in the core bactofilin domain, without any needed co-factors [12]. Point mutations in *C. crescentus bacA* have identified residues that disrupt polymerization *in vivo*, which are important for its localization to the stalked pole in cells [12]. Analogous mutations in *H. pylori* CcmA also disrupt polymerization *in vitro*, result in mislocalization *in vivo,* and produce a morphology indistinguishable from the *Δ*ccmA** mutant [35]. Taken together, these results show that bactofilin polymerization can be easily disrupted by mutating selected residues, and that polymerization is required for the proper localization and function of bactofilins *in vivo*.

Structural analyses of the bactofilin TtBac from *Thermus thermophilus* have further elucidated the molecular interactions involved in bactofilin polymerization. TtBac protofilaments polymerize through a β-stacking mechanism similar to amyloid fibers. Interestingly, the monomers in the protofilament are organized in a non-polar fashion, with alternating N-terminal to N-terminal and C-terminal to C-terminal interactions [8]. The N-terminal tails are thought to mediate inter-protofilament interactions, allowing for the formation of protofilament bundles. Furthermore, a short hydrophobic motif in the N-terminal domain appears to mediate bactofilin binding to lipid membranes *in vitro* and *in situ* [8], consistent with the bactofilins’ ability to recruit proteins to specific sites in the cell envelope *in vivo*.

The domain architecture and polymerization properties of the bactofilins are thought to be highly conserved among bacteria, but it is unclear how they contribute to bactofilin function *in vivo*. Here, we utilize *A. biprosthecum* as a morphogenic system to examine this question, applying mutations that affect its bactofilin BacA’s polymerization and N- and C- terminal domain function. *A. biprosthecum* is an ideal *in vivo* system to examine the functional effects of such mutations, as wild-type stalks and *Δ*bacA** pseudostalks provide easily distinguishable phenotypes. We present data that show that polymerization, as well as the disordered, proline-rich N- and C-terminal domains of BacA, are required for its function; mutations abolishing these features lead to severe defects in stalk synthesis, ranging from fewer and shorter stalks, to full pseudostalk synthesis. Next, we show that these mutations affect the localization dynamics of BacA. These results, along with other disparate lines of evidence, suggest that bactofilins represent an evolutionary Swiss army knife, a common platform (the self-polymerizing central β-helical core domain) upon which various adaptations can be made (using the N- and C-terminal domains) to address different biological functions.

## Results

### Defining the core bactofilin domain and the flanking regions in A. biprosthecum BacA

Bactofilin proteins can self-polymerize, which is important for their function in different bacteria [10]. Bactofilin polymerization is solely dependent on the conserved central bactofilin domain of the protein, whereas the flanking N- and C- terminal domains often vary between genera and species. To gain insight into the domain architecture of *A. biprosthecum* BacA (Abi_34180; hereafter referred to as BacA), we first wanted to precisely map its domains in comparison to *C. crescentus* and other related species in the family *Caulobacteraceae*.

**Fig 1:**
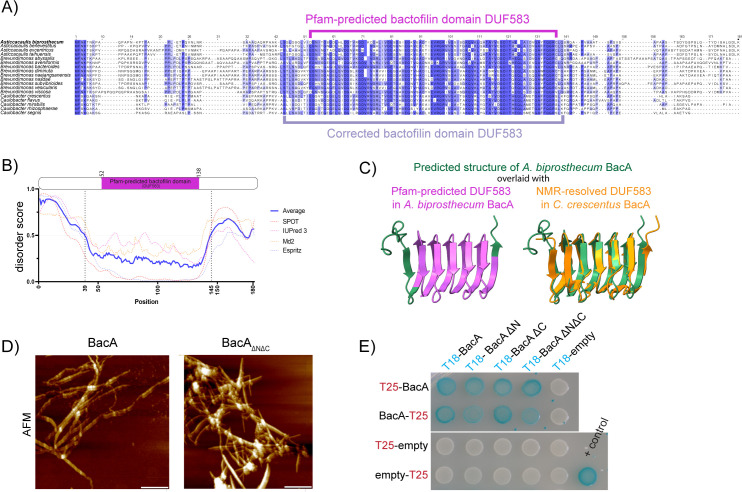
The bactofilin domain of *A. biprosthecum* BacA extends beyond the Pfam- predicted domain sequence, and forms filaments *in vitro* even in the absence of the BacA N- and C- terminal domains. **A)** Multiple sequence alignment of bactofilin sequences from various species from the *Caulobacteraceae* family. The sequences are colored using a percentage-identity color scheme, which color-codes based on conservation (dark blue: above 80%; blue: above 60%; light blue: above 40%). The Pfam-predicted bactofilin domain (DUF583) is indicated with a magenta bracket, and the corrected bactofilin domain with a mauve bracket. **B)** Disorder plot of the *A. biprosthecum* BacA sequence, generated using SPOT-Disorder2, IUPred 3, Md2, and Espritz. Disorder scores were normalized from 0 to 1. Amino acid positions with a disorder score above 0.5 are considered disordered regions of the protein. A schematic of the BacA protein indicating its Pfam-predicted bactofilin domain (DUF583), is aligned with the disorder plot as reference. **C)** Left: Structural superposition of the structure of *A. biprosthecum* BacA predicted using AlphaFold (green), with its Pfam-delimited bactofilin domain (DUF583) in magenta. Right: Structural superposition of the predicted structure of *A. biprosthecum* BacA (green) with the NMR-resolved structure of *C. crescentus* BacA (PDB-ID: 2N3D, orange). **D)** High-resolution AFM images of purified BacA filaments and BacA_ΔNΔC_. Scale bar = 1 µm. **E)** Bacterial two-hybrid (BACTH) assay in which pairs of proteins are fused to T18 and T25 fragments of adenylate cyclase and co-expressed in *E. coli*. Protein pairs that interact *in situ* enable the T18 and T25 fragments to carry out adenylate cyclase activity, which is reported as blue patches on beta-galactosidase culture plates. Here, T18-BacA constructs, with or without the BacA N- and C- terminal domains, are probed against full-length BacA fused to the T25 probe either on its N- or C-terminal. A strain co-expressing T18 and T25 fragments fused to interacting segments of a Leucine Zipper motif was used as a positive control (blue patch, bottom right). Negative controls (white patches) were performed against unfused T25 fragments.

The predicted bactofilin domain (DUF583) of BacA, as determined by bioinformatic Pfam analysis, spans residues 52–138 of the protein. However, this was shorter than the overall sequence conservation observed in the central domain of this protein among closely related species within the Caulobacteraceae family (Fig 1A). Indeed, the Pfam-predicted bactofilin domain in this family excluded several highly conserved amino acids. Moreover, bactofilin domains are usually flanked by proline rich N- and C- terminal domains [9], but none of the conserved residues adjacent to the Pfam-predicted bactofilin domains were prolines (S1A Fig). An examination of BacA with disorder prediction software revealed that residues 39–145 were likely structured, which includes several conserved residues excluded from the Pfam-predicted bactofilin domain (Fig 1B). Upon conducting an AlphaFold structure prediction of BacA and juxtaposing it with the experimentally determined protein structure of *C. crescentus* BacA (_Cc_BacA), it became evident that the current annotation of the bactofilin domain (DUF583) by Pfam falls short of capturing the complete structured bactofilin domain of BacA (Figs 1C, S1B and S2). Notably, the Pfam-predicted bactofilin domain lacks an entire helix at the N-terminal end and the last few amino acids at the C-terminal end of the bactofilin domain (Fig 1C, left). Based on these observations, we predicted that the bactofilin domain of BacA is composed of amino acid (aa) residues 44–140, which match the ordered β-helical core of _Cc_BacA. However, several residues following 140Q exhibit notable conservation among the *Caulobacteraceae*. Consequently, we opted to assess if a bactofilin domain missing these residues would still be able to polymerize properly. To test this, we overexpressed and purified the bactofilin domain encompassing residues 44–140 (hereafter referred to as BacA_∆N∆C_) in *E. coli* and found that it demonstrated successful filament formation *in vitro* (Figs 1D and S1C). This demonstrates that the conserved residues beyond 140Q are unnecessary for polymerization and can thus be excluded from our updated definition of the BacA bactofilin domain. Notably, a shorter stretch of the BacA bactofilin domain (aa 44–138) failed to form filaments and precipitated in solution without any filamentation observed (S1D).

In contrast to the structured bactofilin domain which drives polymerization, the N- and C-terminal domains of bactofilins are predicted to be highly disordered, and their functions are less understood. To investigate their importance, we designed truncation mutations of BacA, annotated as BacA_ΔN_ (Δ2–43), BacA_ΔC_ (Δ141–181), and BacA_ΔNΔC_ (Δ2–43 and Δ141–181), removing either the N- or C-terminal domains or both (S1E Fig). In all cases, the truncated BacA proteins were able to interact with wild-type (WT) BacA in a bacterial adenylate cyclase two-hybrid (BACTH) assay in *E. coli* (Fig 1E).

### Role of the N- and C- terminal domains of BacA in stalk synthesis

**Fig 2:**
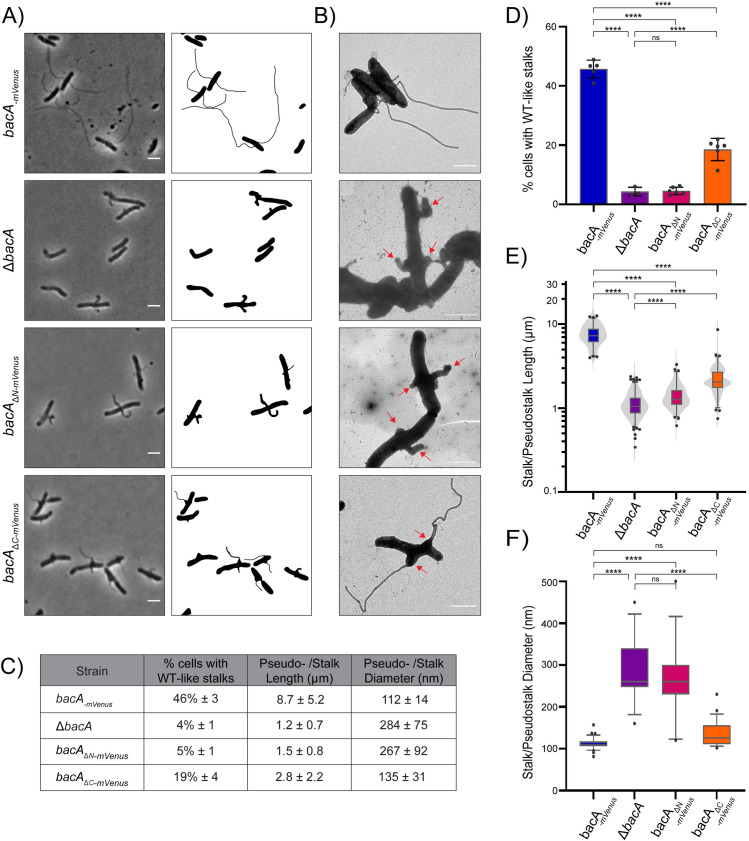
The disordered N- and C- terminal domains of BacA are required for normal morphogenesis of the stalk. **A)** Phase contrast images (left) and corresponding cell silhouettes (right) of *A. biprosthecum bacA-mVenus*, Δ*bacA*, *bacA*_Δ*N*_*-mVenus* and *bacA*_Δ*C*_*-mVenus,* to analyze stalk morphology. Cells were grown in phosphate-limited (HIGG) medium (see Methods). Scale bars = 2 μm. **B)** Transmission electron microscopy of the same strains as in Panel A. Pseudostalks are indicated with red arrows. Scale bars = 2 μm. **C)** Summary statistics for data presented in Fig 2D-F. Data shown as mean ± SD. **D)** Percentage of cells with WT-like stalks. Based on phase images, cells were scored as having a WT-like stalk (i.e., a thin extension from the cell body). Cells exhibiting thick and aberrant pseudostalks were excluded (*bacA-mVenus* n = 3966; Δ*bacA* n = 2749; *bacA*_Δ*N*_*-mVenus* n = 3034; *bacA*_Δ*C*_*-mVenus* n = 3879). Data are presented as the mean percentage of cells with WT-like stalks. Error bars represent SD. (****p ≤ 0.001, ns = not significant p > 0.05; t-test). **E)** Distribution of stalk/pseudostalk lengths in the different strains (*bacA-mVenus* n = 575; Δ*bacA* n = 776; *bacA*_Δ*N*_*-mVenus* n = 448; *bacA*_Δ*C*_*-mVenus* n = 576). Data are represented as box and whisker plots and violin plots. (****p ≤ 0.001; t-test). **F)** Distribution of stalk/pseudostalk base diameter in the different strains based on atomic force microscopy images (*bacA-mVenus* n = 32; Δ*bacA* n = 13; *bacA*_Δ*N*_*-mVenus* n = 15; *bacA*_Δ*C*_*-mVenus* n = 23; see S3B Fig for representative images). Data are represented as box and whisker plots plots. (****p ≤ 0.001, ns = not significant p > 0.05; t-test).

To examine if BacA’s non-polymerizing regions are important for stalk synthesis in *A. biprosthecum*, we created BacA mutants lacking the N- and C-terminal domains, expressed at the native *bacA* locus in *A. biprosthecum*, with an mVenus tag to observe their localization and assess their expression (S3A Fig). Full length BacA-mVenus expressed from the native *bacA* locus was used for comparison, to determine the function of these truncated mutants. This fusion was shown to have no effect on BacA function in a previous publication [32]. Deletion of the entire *bacA* gene or BacA’s N- or C- terminal domains resulted in a pseudostalk phenotype, with most stalked cells producing misshaped protrusions at the site of stalk synthesis (Fig 2A and 2B). The *bacA*_*∆N*_*-mVenus* strain demonstrated a phenotype nearly as severe as *Δ*bacA,** with only 5 ± 1% of these cells forming WT-like stalks, compared to 46 ± 3% in the *bacA-mVenus* strain (Fig 2C and 2D). Instead, most stalked cells of the *bacA*_*∆N*_*-mVenus* strain formed enlarged, short pseudostalks similar in size (1.5 ± 0.8 μm long) and diameter (267 ± 92 nm wide) to *Δ*bacA** pseudostalks (Fig 2E and 2F). Thus, loss of the N-terminal domain of BacA nearly completely abolishes BacA function *in vivo* in terms of regulating stalk synthesis, despite its ability to interact with WT BacA in BACTH experiments*,* and the retained ability of BacA_ΔNΔC_ to self-interact *in vitro* (Fig 1D and 1E). In contrast, absence of the C-terminal domain of BacA led to a less severe phenotype. A greater proportion of *bacA*_*∆C*_*-mVenus* mutants had WT-like stalks (19 ± 4%), while other stalked cells produced pseudostalks with phenotypes intermediate to WT and ∆*bacA* (Fig 2A-2F). The vast majority of WT-like stalks in BacA_∆C_-mVenus cells emerged from a pseudostalk base, from which extruded a prolonged, thin stalk (Figs 2A, 2B and S3B). This suggests that loss of the C-terminal domain of BacA is detrimental to stalk formation, but nonetheless allows partial functionality of the BacA_∆C_ protein.

**Fig 3:**
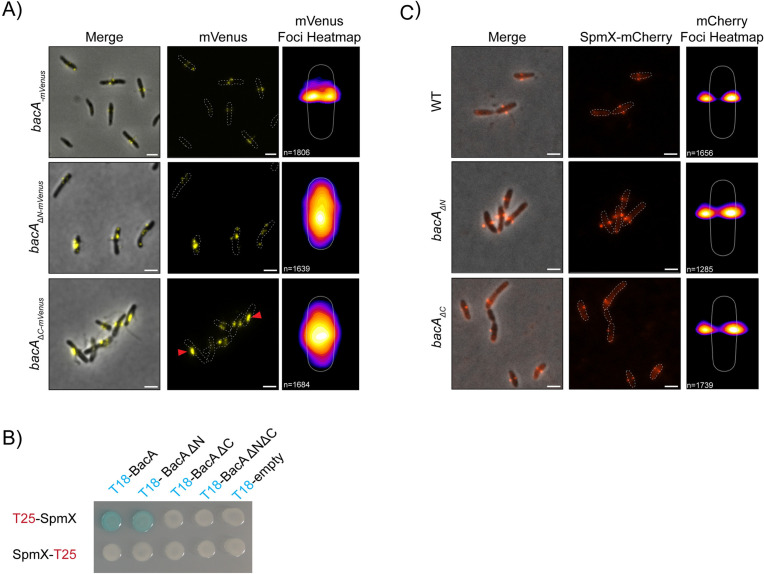
The N- and C-terminal domains of BacA are necessary to localize BacA at the site of stalk synthesis but are not necessary for SpmX localization despite impaired interaction. **A)** Merged phase contrast/fluorescence (left) and fluorescence (middle) microscopy images with localization heatmaps (right) of *A. biprosthecum* WT BacA-mVenus, BacA_ΔN_-mVenus, and BacA_ΔC_-mVenus. Red arrowheads indicate elongated foci of BacA_ΔC_-mVenus. The number of cells analyzed is shown on the bottom left of each heatmap. Scale bars = 2 μm. **B)** Bacterial two-hybrid (BACTH) assays as presented in Fig 1E, with T18-BacA constructs with or without the BacA N- and C- terminal domains, probed against SpmX fused to the T25 probe either on its N- or C-terminal. **C)** Merged phase contrast/fluorescence (left) and fluorescence (middle) microscopy images with localization heatmaps (right) of SpmX-mCherry in *A. biprosthecum* WT, *bacA*_*ΔN*_, and *bacA*_*ΔC*_ strains grown in 90 µM phosphate HIGG. The number of cells analyzed in each case is shown on the bottom left of each heatmap. Scale bars = 2 μm.

Next, we examined the localization of the tagged BacA mutant constructs. Whereas WT BacA-mVenus localized as one or two discrete, bilateral foci at the midcell, consistent with the sites of stalk synthesis, BacA_ΔN_-mVenus no longer showed bilateral foci (Fig 3A). Instead, BacA_ΔN_-mVenus foci were weaker and could be observed throughout the cell except at the poles, sometimes falling near the site of pseudostalk synthesis. In addition, BacA_ΔN_-mVenus cells were more likely to be without foci (25% of cells, compared to 11% in WT BacA-mVenus (S4B Fig)). In contrast, BacA_∆C_-mVenus showed a more intermediate phenotype, localizing laterally at the membrane, but forming single elongated foci that may correspond to BacA_∆C_-mVenus filaments (red arrowheads, Fig 3A), perhaps from an accumulation of the protein due to its failure to consistently localize to the stalk synthesis site. Nevertheless, whenever a WT-like stalk or an intermediate stalk phenotype was observed in this strain, BacA_ΔC_-mVenus foci were always present at or near the base of these stalks.

In *A. biprosthecum,* the developmental regulator of stalk synthesis, SpmX, is required for localization of BacA to the base of the stalk. Conversely, BacA functions to stabilize SpmX at the stalk base, and the two proteins interact directly ([32] and S3C Fig). To test whether the N- or C-terminal domains of BacA are involved in interactions with SpmX, we conducted a BACTH assay in *E. coli* and found that loss of the C-terminal domain of BacA led to a loss of SpmX-BacA interactions, while deletion of the N-terminal domain did not (Fig 3B). We also tested whether SpmX recruitment and localization were impacted in untagged *bacA* mutant strains, by expressing SpmX-mCherry in these backgrounds. SpmX localizes to the stalk base in WT cells [28]; however, in the *bacA*_*∆C*_ and *bacA*_*∆N*_ mutants, we observed that SpmX-mCherry localizes within the stalk or pseudostalk structures, while mostly displaying characteristic bilateral foci (S4A Fig). This suggests that the bilateral localization of SpmX is preserved in *bacA*_*∆N*_ and *bacA*_*∆C*_, with their altered localization compared to WT likely being a consequence of pseudostalk formation, as previously observed in *∆bacA* [32]. To further validate this observation, we examined SpmX localization independently of stalk morphology, under higher phosphate concentrations where stalks and pseudostalks are less prominent. Under these conditions, we found that the distribution of SpmX-mCherry foci was not impacted in the *bacA*_*∆N*_ and *bacA*_*∆C*_ mutants, in both of which the foci localized bilaterally at the stalk base as in WT cells (Figs 3C and S4B). However, we noticed a small increase in cytoplasmic SpmX-mCherry fluorescence in all *bacA*_*∆C*_ cells compared to WT (Fig 3C). Furthermore, as BacA_ΔC_ shows intermediate phenotypes for both stalk formation and localization (Figs 2 and 3A), we wanted to test whether SpmX and BacA_ΔC_ colocalize during stalk formation in this mutant. In co-localization experiments, we found that BacA_ΔC_-mVenus did not colocalize with SpmX-mCherry in most cells. However, BacA_ΔC_-mVenus could be found in the vicinity of the stalk site (arrow, S4C Fig), implying that a compensatory recruitment mechanism may allow this mutant to rescue normal stalk synthesis in some cells.

### Role of bactofilin polymerization in stalk synthesis

**Fig 4:**
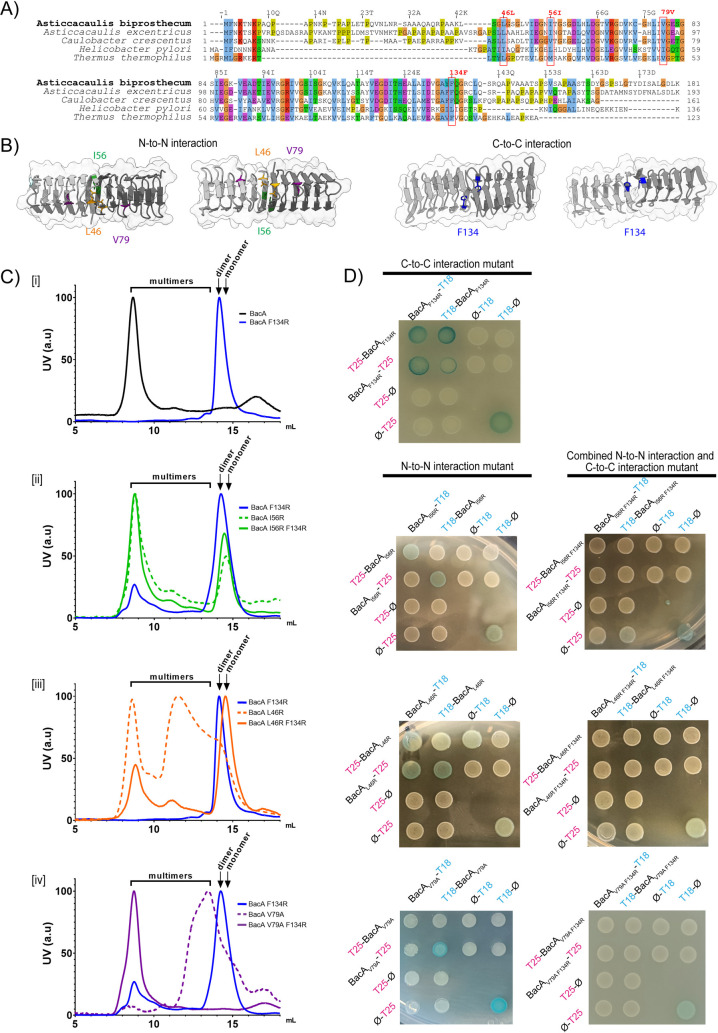
Various amino acid residues in the N-to-N and C-to-C interfaces are implicated in BacA polymerization. **A)** Multiple sequence alignment of bactofilin sequences from *A. biprosthecum*, *A. excentricus*, *C. crescentus, H. pylori*, and *T. thermophilus*. The alignment is colored using the Clustal X color scheme, which color codes amino acid residues with shared properties. The red boxes indicate conserved amino acids that have been shown to be important for bactofilin polymerization in other species, selected for substitution in *A. biprosthecum* in this study. **B)** Structural representation of the BacA dimer derived from the BacA tetramer structure predicted using AlphaFold, showing either the N-to-N interface (left) or the C-to-C interface (right). Mutated residues are highlighted. **C)** Analytical size exclusion chromatography of purified BacA carrying various mutations. Elution profiles are color coded as follows - Black: WT BacA; Blue: BacA_F134R_; Green: BacA_I56R_; Orange: BacA_L46R_; Purple: BacA_V79R._ Single N-to-N interface mutants are displayed with dashed lines while double F134R and N-to-N interface mutants of BacA are displayed as solid lines. Subpanels [i] to [iv] highlight different constructs: [i] F134R; [ii] I56R, [iii] L46R, [iv] V79A. Curves are normalized such that the maximum intensity peak is 100. The expected elution ranges of monomers, dimers and multimers are represented above each curve. **D)** *E. coli* BACTH assay with T18-conjugated BacA constructs mutated in one (left) or both (right) polymerization interfaces, probed against the same constructs conjugated to T25, fusing each probe to either the N- or C-terminal of the BacA protein. Negative controls were performed against unfused T25 fragments. Positive controls (+) are shown on the bottom right of each plate.

To assess the impact of bactofilin polymerization on stalk formation, we aimed to identify key residues at both the N-to-N and C-to-C polymerization interfaces of the core domain. We referred to previous studies elucidating the effects of point mutations on bactofilin polymerization in other species [8,12,36], and derived insights from sequence conservation among BacA homologs as well as from structural predictions of the *A. biprosthecum* bactofilin domain (Figs 4A, 4B and S2). In other species, the most impactful substitution disrupting higher-order polymerization was in the C-to-C interface of the bactofilin domain: F130A in *C. crescentus*, L110S in *H. pylori*, and F105R in *T. thermophilus* [8,12]. All of these residues align with *A. biprosthecum* F134, which we substituted with an arginine for testing (Fig 4A and 4B). When we overexpressed and purified the BacA_F134R_ mutant protein in *E. coli,* we were unable to detect filament formation or higher-order structures via atomic force microscopy (S5C Fig). Size-exclusion chromatography data showed that purified BacA_F134R_ eluted in a single peak at around 14 mL, corresponding to a globular protein of 45 kDa, which corresponds to the size of a BacA dimer (blue line, Fig 4Ci). Since bactofilin filaments are known to polymerize in a non-polar manner (i.e., alternating N-to-N and C-to-C interactions), our data suggest that the BacA_F134R_ mutation may disrupt C-to-C interactions, preventing multimerization, while preserving N-to-N interactions, allowing dimerization.

In the N-to-N interface of the bactofilin domain, multiple residues have been shown to affect bactofilin polymerization in other species. Notably, *C. crescentus* V75 (*H. pylori* I55), which corresponds to V79 in *A. biprosthecum,* is important for *in vivo* and *in vitro* filament assembly [12,35]. However, this residue does not lie directly at the N-to-N interface (Fig 4B), suggesting that its mutation may affect BacA monomer structure more broadly, resulting in an altered N-to-N interface. To test the importance of this residue in *A. biprosthecum,* we constructed a BacA_V79A_ mutant. We also mutated L46, predicted to lie at the N-to-N interface, whose substitution to a serine in *C. crescentus* showed fainter and larger _Cc_BacA_L46S_-mVenus foci and an increase in cell body fluorescence compared to WT [12]. However, as serine is relatively small and only weakly hydrophilic, this substitution may not completely disrupt _Cc_BacA polymerization dynamics. Therefore, we constructed an L46R substitution. Additionally, we selected another conserved hydrophobic residue, I56, located at the N-to-N interface but on the opposite side of the L46 residue (Fig 4B), creating an I56R substitution to test its role in polymerization.

In size-exclusion chromatography, the BacA_I56R_ mutant eluted mostly at around 9 mL, similar to purified WT BacA (Fig 4Cii, dashed green line). On the other hand, BacA_L46R_ and BacA_V79A_ eluted mostly at 10–14 mL, although BacA_L46R_ also showed a significant peak at around 9 mL, similar to WT BacA (Fig 4Ciii and 4Civ, dashed orange and purple lines). However, the observed multimeric peaks of these mutant proteins (at 9 mL) could correspond to BacA filaments or to unfolded protein aggregates. To distinguish between these possibilities, we examined their polymerization capacity by atomic force microscopy (AFM). Importantly, we found that the L46R and V79A mutants did not form filaments; instead, we observed aggregates that likely resulted from spontaneously unfolded proteins (S5C Fig). However, the BacA_I56R_ mutant was able to polymerize *in vitro,* forming structures characterized by short polymer lengths but with unusual width (ranging from 40 to 220 nm, compared to 9–60 nm typical for WT). This suggests that the I56R mutant may have a unique polymerization mechanism distinct from the wild-type protein. Consistent with the above results, these individual N-to-N interface mutants always retained some self-interaction in at least one configuration in a BACTH assay (Fig 4D), likely explained by their retention of the C-to-C polymerization interface.

To test mutations affecting both the N-to-N and C-to-C polymerization interfaces simultaneously, we created strains carrying the N-to-N interface substitutions in combination with the F134R substitution at the C-to-C interface. Mutating the N-to-N interface with either the I56R or L46R substitution in combination with the F134R substitution in the C-to-C interface resulted in a larger peak eluting at around 15 mL, corresponding to 22 kDa, the size of a BacA monomer (Fig 4Cii and 4Ciii, solid green and orange lines), consistent with a disruption of both polymerization interfaces. However, both BacA_I56R F134R_ and BacA_L46R F134R_ still showed a peak at around 9 mL, corresponding to the elution time of the WT BacA multimer. Nevertheless, in the BACTH assay, BacA_L46R F134R_ and BacA_I56R F134R_ did not display self-interaction (Fig 4D), suggesting that the multimers in their chromatography are aggregates of misfolded proteins, as confirmed by AFM (S5C Fig). Similar aggregation was seen when the V79A substitution was made in combination with the F134R substitution; BacA_V79A F134R_ produced a multimeric peak at around 9 mL similar to BacA_L46R F134R_ and BacA_I56R F134R_ (Fig 4Civ, solid purple line), but only showed protein aggregates in AFM (S5C Fig).

**Fig 5:**
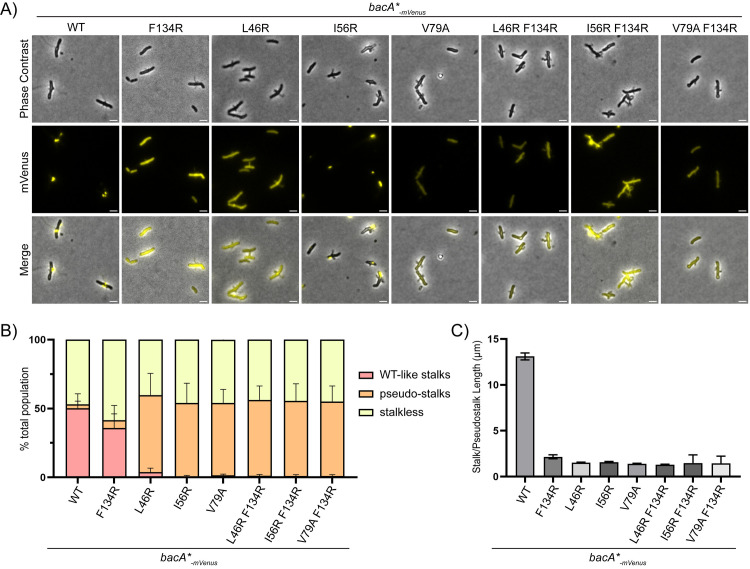
The BacA_F134R_ C-to-C interface polymerization mutant retains some functionality for stalk synthesis *in vivo* in *A. biprosthecum.* **A)** Phase contrast, fluorescence and merged microscopy images of BacA-mVenus strains carrying various polymerization mutations, grown in phosphate-limited HIGG medium. For better visualization, LUTs were adjusted to reduce the brightness of the BacA-mVenus and BacA I56R-mVenus fluorescence signals, as their punctate localization showed much higher intensity compared to the diffuse signals in other strains. See S5B Fig for fluorescent images of these two strains with the same LUTs as the other mutants presented in this figure.Scale bars = 2 μm. **B)** Percentage of cells with different types of stalks for each of the BacA-mVenus polymerization mutant strains. Based on phase contrast images from three independent biological replicates, stalks were assessed and categorized either as WT-like stalks or pseudostalks. Data are presented as the mean percentage of cells with WT-like stalks or pseudostalks. Error bars represent SD. Total numbers of cells for each strain were as follows: WT n = 1940; F134R n = 1312; L46R n = 1817, I56R n = 1645; V79A n = 1483; L46R F134R n = 1308; I56R F134R n = 1907; V79A F134R n = 1746. **C)** Distribution of stalk/pseudostalk lengths in BacA-mVenus polymerization mutant strains. Error bars represent SD. Total numbers of cells for each strain were as follows: WT n = 189; F134R n = 63; L46R n = 192, I56R n = 113; V79A n = 167; L46R F134R n = 155; I56R F134R n = 38; V79A F134R n = 70).

Finally, we wanted to determine the effects of these polymerization mutations on BacA localization and stalk synthesis in *A. biprosthecum*. We examined this in *A. biprosthecum* using mVenus-tagged constructs of BacA carrying the different point mutations. Testing the expression of these mutant proteins in Western blots using anti-mVenus antibodies, we found that most of the mutations resulted in a reduced amount of BacA in the cell (S5A Fig). Fluorescence microscopy showed a diffuse cell body fluorescence for most of these mutants, as opposed to the discrete bilateral foci seen with WT BacA-mVenus (Fig 5A). Only one of these mutants did not have diffuse cytoplasmic fluorescence; BacA_I56R_-mVenus showed large, delocalized foci, consistent with the ability of this mutant to form polymers *in vitro*. This suggests that the I56R mutation may impair BacA localization to a lesser extent than the other point mutations (Fig 5A). Notably, BacA_I56R_-mVenus localization was reminiscent of BacA_ΔN_- or BacA_ΔC_-mVenus localization (Fig 3A), indicating that it retains some ability to form larger bactofilin structures *in vivo,* though with significantly impaired precision of localization compared to WT BacA-mVenus.

Next, we used phase contrast microscopy to assess stalk formation and stalk length in the different polymerization mutants. Given that the C-to-C polymerization interface mutant BacA_F134R_ lacks the ability to polymerize beyond a dimer, we expected it to lead to impaired stalk formation. But surprisingly, we observed stalks in 41% of BacA_F134R_ cells (compared to 50% in WT) (Fig 5B). Most of the stalks were short and thin (Fig 5A), meaning that impairment of C-to-C polymerization does not completely inhibit stalk formation, but restricts them in terms of length (Fig 5C). In contrast, all N-to-N interface mutants had pseudostalks, both by themselves and in combination with the F134R mutation, including the I56R mutant despite its partial ability to polymerize and localize (Figs 5A and S5C). In these strains, pseudostalks were observed in a similar proportion of cells as stalked cells in the WT background (Fig 5A and 5B). Their pseudostalks were highly reminiscent of the *∆bacA* phenotype, similar in proportions and stalk lengths, and resulting in the same sorts of cell abnormalities as previously described for *∆bacA* [29].

## Discussion

Over the past few decades, it has become evident that filament-forming cytoskeletal proteins are important for bacterial cellular organization [1,2]. Among these, bactofilins, a relatively recently discovered cytoskeletal filament, are highly conserved, widely distributed, and implicated in a variety of cellular processes including morphology [7,9,14,15,22,31]. The bactofilin proteins studied thus far share the common characteristics of 1) a conserved β-helical core (the bactofilin domain) flanked by variable N- and C-terminal regions and 2) the ability to self-polymerize without requiring cofactors. Using *A. biprosthecum* as a model system, we were able to show that these disordered N- and C-terminal regions are important for BacA’s role in stalk synthesis. Moreover, we showed that BacA polymerization is also important for its role in stalk synthesis, relying on critical residues at both the N-to-N and C-to-C polymerization interfaces.

In many organisms, bactofilins act as a morphogenesis protein through interactions with peptidoglycan-associated proteins. Often, they are found in the same operon as a peptidoglycan hydrolase LmdC, which has been found to be important for morphogenesis in *H. neptunium* and *Rhodospirillum rubrum* [23]. In *H.* *pylori*, the bactofilin CcmA interacts with various Csd (cell shape determining) proteins. In *C. crescentus* the bactofilins BacA and BacB interact with the PG-synthetic protein PbpC [9,15]. The presence of proline-rich domains in both the N- and C-terminal domains of bactofilins may facilitate their versatile interactions with other morphogenic proteins, potentially allowing them to regulate peptidoglycan synthesis akin to what has been observed with other peptidoglycan-related scaffolding proteins.

In recent years, the N-terminal domains of bactofilins have been implicated in membrane binding. In a limited subset of Gammaproteobacteria*,* bactofilins even have transmembrane domains within their N-terminal regions, although most bacteria instead rely on a short amphipathic peptide sequence for membrane associations [8,37,38]. In our study, we observed that deleting the N-terminal region of BacA led to a pseudostalk phenotype as severe as observed with deletion of the entire *bacA* gene. Using an mVenus-tagged fusion, we found that BacA_∆N_ was still able to form foci *in vivo,* but we found that these foci were mislocalized throughout the cell. This is in contrast to what has been found in *C. crescentus*, where mutations in the N-terminal membrane-binding region, result in a diffuse localization of _Cc_BacA underscoring a potential interdependence between polymerization and membrane binding [38]. It is possible that this difference is due to the different recruitment role played by SpmX in the *Asticcacaulis* genus [28].

In *A. biprosthecum*, SpmX is responsible for the recruitment of the stalk synthesis machinery, including BacA, to the base of the stalk. While interactions between SpmX and BacA_∆N_ were not lost in a BACTH assay, SpmX nonetheless does not seem to be able to recruit BacA_∆N_ to the base of the stalk, as seen by the general absence of BacA_∆N_-mVenus foci at the stalk base, and its pseudostalk phenotype reminiscent of a *∆bacA* mutant [32]. Meanwhile, deletion of the N-terminal domain of BacA had no detectable effect on SpmX localization, even though BacA_∆N_ is not localized at the stalk base. Based on these results, we hypothesize that the N-terminal domain of BacA, perhaps through its interactions with the membrane, plays a role in its recruitment to the base of the stalk. Loss of the N-terminal domain of BacA thereby prevents its localization at the stalk base leading to a pseudostalk phenotype. In *H. pylori*, CcmA’s short N-terminal region binds to the the bactofilin core domain and acts as an inhibitory domain which prevents interaction with Csd7, thereby regulating protein complex formation and stability [20]. Our structural prediction models (S2 Fig) suggest that the N-terminal domain does not interact with the bactofilin core domain which could indicate that the CcmA and BacA N-terminal domains function through distinct mechanisms. BacA’s N-terminal domain is significantly longer (43 aa) and contains a proline-rich region that could facilitate protein-protein interactions directly to stabilize BacA at the stalk site.

Shifting focus to the C-terminal domain of bactofilins, previous studies suggest that it plays an important role in protein-protein interactions involving the bactofilins. In *M. xanthus*, the C-terminal domains of its bactofilins interact with the PadC-ParA chromosome segregation complex [19]. In this species, fluorescent tagging on the C-terminus of the bactofilins failed to give proper localization or complement gene deletion, likely due to molecular crowding from the fluorescent proteins preventing its protein-protein interactions [18,19]. Similarly, in *P. mirabilis*, a transposon insertion at the C-terminus of the bactofilin CcmA caused a more severe morphological phenotype than gene deletion, indicating that such an insertion disrupts other functions in the cell [16]. These observations underscore the crucial role of the C-terminal domain in bactofilin activity, particularly for protein-protein interactions. In *C. crescentus, Liu et al.* demonstrated that BacA-PbpC interaction occurs at the C-terminal interface of the core domain, indicating that BacA can engage in protein interactions using multiple domains rather than exclusively through the C-terminal region [38]. PbpC lacks homologs in the *Asticcacaulis* genus, and the specific PG synthetic enzymes required for stalk synthesis have not yet been identified in *A. biprosthecum*. Here, we determined that the BacA C-terminal domain deletion disrupts bacterial two-hybrid interactions with SpmX, which in turn may be important for BacA recruitment to the stalk synthesis site. In the absence of the C-terminal domain, BacA_∆C_-mVenus mislocalizes, preventing its recruitment to the base of the stalks as discrete foci, in contrast to WT BacA-mVenus. Interestingly, BacA_∆C_ cells produce multiple phenotypes ranging from WT-like stalks to ∆*bacA*-like pseudostalks. Specifically, many cells show a widened stalk base extruding thin stalks at their tips. This phenotype is associated with elongated BacA_ΔC_-mVenus foci at the base of these thin stalks (either at the regular stalk base or within the larger pseudostalk-like base of the extended thin stalk). Like DivIVA or MreB, Bactofilins in various organisms accumulate at sites of specific membrane curvature [7,20,35,39,40]. BacA recruitment could depend on membrane modifications at the stalk site following SpmX recruitment. The ability of SpmX to form multimers *in vitro* supports this possibility [41], as multimerization imposes mechanical stress on the membrane that could form sub-micron curvature, allowing BacA recruitment or stabilization. In the absence of the C-terminal domain, the initial round of peptidoglycan insertion in a pseudostalk-like manner could create detectable membrane curvature, which could then facilitate BacA_ΔC_ recruitment to the stalk site through the N-terminal domain of the protein. Alternatively, BacA_ΔC_ may interact with other stalk synthesis proteins recruited by SpmX at the stalk base, allowing rescue of WT-like stalk synthesis. This suggests that 1) the SpmX-BacA interaction is not required throughout the entire stalk synthesis process and 2) the C-terminal domain is not the only domain mediating BacA’s protein-protein interactions for stalk synthesis, which may also be mediated through the core bactofilin domain or the N-terminal domain. This was observed in *H. pylori* where the C-terminal domain does not contribute to cell shape maintenance and all known CcmA-Csd protein interactions occur through the remaining domains [15,20,35].

Bactofilins have been characterized by the presence of the DUF583 domain, i.e., the bactofilin domain, comprising a β-helical structure with a hydrophobic core [8,12]. Bactofilins polymerize via their hydrophobic domain interactions to form filaments and *in vivo* structures. The critical role of bactofilin polymerization is supported by findings in other species, where polymerization disruptions lead to significant functional impairments. Here, we mutated specific hydrophobic residues predicted to mediate interactions at the N-to-N and C-to-C interfaces to test the importance of BacA polymerization for its function in *A. biprosthecum*.

As reported previously, we confirmed that BacA forms non-polar filaments based on BACTH and size-exclusion chromatography data. We were also able to construct a fully monomeric bactofilin by mutating both the N-to-N and C-to-C polymerization interfaces with the L46R and F134R mutations. When we tested other N-to-N interface mutations for their effects on polymerization, we surprisingly found that the I56R mutant could form filaments *in vitro*, albeit with shorter length and greater thickness than wild-type filaments. Furthermore, BacA_I56R_-mVenus also formed foci *in vivo* that were distributed throughout the cell body – the only polymerization point mutant to do so (all others showed diffuse cell body fluorescence consistent with their inability to polymerize). Interestingly, the equivalent mutation of I56R in *C. crescentus*, V52A, has also been shown to form polar foci [12], suggesting that the N-to-N interface requires greater disruption than achieved by these substitutions. Alternatively, other polymerization mechanisms may come into play when end-to-end interactions (i.e., C-to-C and N-to-N interactions) are compromised in the bactofilins. Indeed, lateral interactions between bactofilin filaments have been observed *in vitro,* where filaments bundle together. While lateral interactions have not been directly demonstrated *in vivo*, their existence remains a possibility that requires further studies to investigate.

When we examined the effect on stalk synthesis of amino acid substitutions that disrupt polymerization, we expected non-polymerizing mutants to completely phenocopy the *∆bacA* mutant, i.e., that they would produce pseudostalks irrespective of the mutated interface, since they should not be able to form the higher-order structures that allow bactofilin function as a stalk-specific scaffolding protein. To our surprise, while the F134R mutation on the C-to-C interface leads to dimer formation *in vitro* and diffuse localization *in vivo*, stalk synthesis was not completely abolished in this strain. Instead, BacA_F134R_ cells appear to produce mostly WT-like stalks, albeit shorter in overall length. Out of all the polymerization mutants we tested, the F134R mutation on the C-to-C interface was the only one showing this phenotype *in vivo.* In contrast, all other polymerization mutants, which targeted the N-to-N interface, including the I56R mutant, which - despite retaining some polymerization and localization ability - still exhibited a *∆bacA*-like pseudostalk phenotype. Based on these findings, we conclude that BacA_F134R_ dimers, which maintain the N-to-N interface, are sufficient for initiation of stalk synthesis, allowing the formation of short WT-like stalks This suggests that stalk synthesis does not strictly require BacA polymerization. However, efficient stalk elongation to produce longer stalks may require a more stable scaffold for PG synthesis, formed by longer BacA polymers. This aligns with previous observations in *C. crescentus,* in which _Cc_BacA/B deletions produce shorter stalks but do not abolish stalk synthesis [9]. Furthermore, interactions between _Cc_BacA and the PG synthase PbpC were also found to be dependent on _Cc_BacA polymerization [38]. Similarly, in the stalked budding bacterium *H. neptunium*, the F130R mutant forms partial stalks but fails to generate budding daughter cells [23], showing that partial stalk formation is possible without full BacA polymerization..

Taken together, our results provide new insights into the division of labor between SpmX and BacA during *A. biprosthecum* stalk morphogenesis. While SpmX plays a vital role in the recruitment of the PG synthesis machinery to the stalk base, our findings suggest that BacA acts as a scaffold to promote the continued coordination and functionality of this machinery during stalk elongation. Specifically, the N- and C-terminal domains of BacA may serve to anchor BacA to the membrane and may influence membrane organization and curvature as observed for archaeal bactofilins [7], while also bringing it in association with SpmX and the stalk synthesis machinery. Thereafter, we propose that BacA polymerization by the core domain promotes the formation of a stable cytoskeletal scaffold to guide efficient peptidoglycan insertion and stalk elongation. Further studies will be needed to elucidate how BacA acts at the base of the stalk to organize PG synthesis machinery and whether it performs other functions such as serving as a diffusion barrier between the stalk and the cell body. Finally, from a broader perspective, we find that while the conserved β-helical core domain is vital for bactofilin polymerization and functionality, the flanking domains may contribute to species-specific functions, reflecting modular evolution of these proteins. The N- and C- terminal domains of the bactofilins vary greatly in sequence and length among species. Considering the diverse mechanisms through which bactofilins appear to be acting and interacting, it is conceivable that they serve as a versatile platform for the generation of novel functions through terminal domain modifications to achieve different scaffolding properties and regulatory interactions in different organisms.

## Methods

### Bacterial strains and growth conditions

All freezer stocks were maintained in 10% DMSO at -70°C*. A. biprosthecum* strains used in this study were grown at 26°C in Peptone Yeast Extract (PYE) medium on plates or in liquid culture, supplemented with 5 μg ml^-1^ kanamycin where appropriate. For microscopy of cells grown in 30 µM phosphate Hutner base-imidazole-buffered-glucose-glutamate (HIGG) medium [42], strains from PYE liquid cultures were washed 2X with double distilled H_2_O before being added in a 1:20 ratio in 30 µM phosphate HIGG, and grown at 26°C with shaking for 48h before imaging. Cells for imaging SpmX localization under higher phosphate conditions were grown in 90 µM phosphate HIGG for 24 hours prior to microscopy.

*E. coli* strains used in this study were grown in liquid lysogeny broth (LB) medium at 37°C (or 26°C for BACTH assays) supplemented with antibiotics or supplements as necessary (carbenicillin 100 µg ml^-1^, kanamycin 50 µg ml^-1^, X-gal (5-Bromo-4-chloro-3-indolyl β-D5-Bromo-4-chloro-3-indolyl β-D-galactopyranoside-galactopyranoside) 40 µg ml^-1^, 0.3 mM diaminopimelic acid and 0.5 mM IPTG).

Mating and electroporation of *A. biprosthecum* were performed as previously described [32]. Outgrowth was performed for 8-24h at 26°C before plating. Mating of plasmids into *A. biprosthecum* was performed using the *dap*‾ *E. coli* strain WM3064 (YB7351) [43]. Allelic exchange and deletions were achieved using a two-step sucrose (3% final) counter selection procedure with pNPTS138. In-house stocks of chemically competent DAP auxotroph (YB7351) and BTH101 (YB9171) *E. coli* cells were prepared as previously described [32]. Plasmids were introduced into *E. coli* strains using chemical transformation according to the manufacturer’s protocols. All strains used in this study can be found in S1 and S2 Tables.

### Recombinant DNA methods

DNA amplification, Gibson cloning, and restriction digests were performed according to the manufacturers’ instructions, using iProof High-Fidelity DNA Polymerase (Bio-Rad Laboratories, Cat. No. 1725302) and NEBuilder HiFi DNA Assembly Master Mix (New England Biolabs, Cat. No. E2621X). All primers for Gibson assembly were designed using NEBuilder Assembly Tool (New England Biolabs) and are available upon request. A detailed list of plasmids can be found in S2 Table. Restriction enzymes and the Gibson cloning mix were purchased from New England Biolabs. Cloning steps were carried out in *E. coli* (alpha-select competent cells, Bioline) and plasmids were purified using GeneJET Plasmid Miniprep Kit (ThermoFisher). Sanger sequencing was performed by the Indiana Molecular Biology Institute (Indiana University) or the IRIC sequencing platform (Université de Montreal) with double stranded plasmid or PCR templates, which were purified with a GeneJet PCR purification kit (ThermoFisher). Whole plasmid sequencing was performed by Plasmidsaurus using Oxford Nanopore Technology with custom analysis and annotation.

### BACTH interaction assays

pUT/pUTc18 fusion and pKT/pKNT25 fusion plasmids containing *bacA,* its mutant constructs, or *spmX* were transformed into BTH101 *E. coli* cells and selected on LB plates with carbenicillin and kanamycin. Colonies were grown overnight in liquid LB with antibiotics and spotted on plates containing X-Gal and IPTG. After overnight incubation at 26°C, plates were imaged using a smartphone camera. Bacterial two-hybrid assays were repeated as previously done in Caccamo et al. [32]. Interaction between T25-SpmX and T18-BacA was detected at 26°C but not at 30°C, whereas no interaction was observed with BacA-T18 at either temperature (S3C Fig).

### Light microscopy and fluorescence imaging

For light microscopy analysis, cells were spotted onto pads made of 1% SeaKem LE Agarose (Lonza, Cat. No. 50000) in HIGG and topped with a glass coverslip. Images were recorded with inverted Nikon Ti-E or Ti-2 microscopes with CFP/YFP/mCherry filter cubes using either 1) a Plan Apo 60X 1.40 NA oil Ph3 DM objective and an iXon X3 DU885 EMCCD camera or 2) a Plan Apo λ 100X 1.45 NA oil Ph3 DM objective and either a Photometrics Prime 95B sCMOS camera or an Andor iXon3 DU885 EM CCD camera.

### Image analysis

Stalk length and stalk percentage data were obtained using FIJI (Fiji Is Just ImageJ). Briefly, phase micrographs were imported into FIJI and stalks were manually traced using the Freehand Line tool and stalk lengths were determined from the manual trace using the Measure function. Data were then plotted on a log10 scale on the y-axis and represented as box and whisker plots where the middle line represents the median, the lower and upper hinges correspond to the first and third quartiles (the 25th and 75th percentiles) and outliers outside 1–99% are plotted individually. A mirrored-density violin plot is underlaid to show the continuous distribution of the data. Percentages of stalked cells per image were calculated by manually counting the number of stalked and non-stalked cells per image. Cells were classified as stalked cells when a distinct extension protruded from the main cell body. Stalk width measurements were made on Atomic Force Microscopy (AFM) height images using Nanoscope software (Bruker, Santa Barbara, CA).

Subcellular localization heatmaps were generated using the ImageJ plugin MicrobeJ (version 5.13o/p) [44]. Detection of foci was performed using adjusted thresholds in MicrobeJ. Threshold values were identical for all mutant strains to ensure comprehensive detection of all foci, which were manually checked to ensure that each focus was only counted once per analysis. Cell silhouettes and cell body outlines were produced by importing phase micrographs into Illustrator (Adobe Inc.) to manually trace the silhouette or outline.

### Bioinformatic analyses and AlphaFold predictions

Genomic and protein sequence data were obtained from the Integrated Microbial Genomes (IMG) database [45]. Multiple sequence alignment of bactofilin sequences was performed using Jalview [46]. Disorder plots were generated using SPOT-Disorder2 [47], IUPred 3 [48], Md2 [49], and Espritz [50]. All were normalized and combined to assemble a single graph to predict BacA’s disordered domains. Structures of the BacA monomer and tetramer were predicted using AlphaFold-multimer as implemented through ColabFold [51]. All predicted structures for the BacA monomer or tetramer can be found in S2 Fig, along with their predicted aligned error scores. Rank 1 predictions were used. For the BacA tetramer, only the core bactofilin domain was used for prediction. Protein structures were visualized and superimposed using ChimeraX [52].

### Protein purification

Recombinant plasmids overproducing His6-BacA with or without mutations were transformed into the BL21 *E. coli* strain. Transformants were grown at 37°C until OD_600_ = 0.6 in LB medium with kanamycin (50 μg ml^−1^). His6-BacA protein expression was induced for 3h at 30°C by adding 0.5 mM IPTG (isopropyl β-D-thiogalactopyranoside). Cells were centrifuged, and pellets were resuspended in 1/25 volume of Buffer A (50 mM HEPES at pH 8, 300 mM NaCl, 2 mM β-mercaptoethanol) and lysed by sonication (20 sec ON/ 40 sec Off, 5 min, Misonix S4000). Cell debris was pelleted by centrifugation at 36,000 g for 30 min at 4°C. The supernatant was loaded onto a Ni-NTA resin (Qiagen) column in an AKTA FPLC pure system. After washing with Buffer A, His6-BacA protein was eluted with a linear gradient of Buffer B (50 mM HEPES at pH 8, 300 mM NaCl, 2 mM β-mercaptoethanol, 500 mM imidazole). Elution fractions were loaded onto SDS page gels and the peak fractions containing the His6-BacA protein were pooled.

Upon purification, His6-tagged WT and mutant BacA proteins were used for a polymerization assay performed by dialyzing the purified protein in Buffer C (25 mM HEPES at pH 8, 250 mM NaCl, 2 mM β-mercaptoethanol). Purified proteins were used for AFM, electron microscopy, or phase contrast microscopy and size-exclusion chromatography.

### Size-exclusion chromatography

500 µl of purified proteins from the polymerization assay were loaded onto a Superdex 200 Increase 10/300 GL column (GE Healthcare) using an AKTA FPLC pure system. Protein elution was monitored using UV and normalized to 100% of the highest observed elution. The column was calibrated with standard protein controls to correlate elution time/volume to protein molecular weight.

### Atomic force microscopy

10 µl of cell suspensions (for whole cell imaging) or diluted proteins (for filament formation) were applied to glass coverslips and left to dry for 2 hours. The dried cells or proteins were imaged using a Bioscope Resolve AFM (Bruker, Santa Barbara, CA) atomic force microscope using contact mode in air.

### Electron microscopy

10 µl of cell suspension was applied to an electron microscopy grid (Formvar/Carbon on 300 mesh; Ted Pella Inc., Cat. No. 01753-F) for 5 min at room temperature. Excess liquid was removed with Whatman filter paper. Cells were then negatively stained with 10 µl 1% uranyl acetate (UA) and excess UA liquid was immediately removed with Whatman filter paper. Grids were allowed to dry, stored in a grid holder in a desiccation chamber, and imaged with a kV JEOL JEM 1010 transmission electron microscope (JEOL USA Inc.).

### Western blot analysis

All strains were grown until OD = 1.0 before being centrifuged and resuspended in 50 μl of 50mM Tris, 200 mM NaCl supplemented with 0.1 μl of Universal Nuclease (Pierce #88700) added with Laemmli buffer. 15 μl of each sample was loaded onto 4%–20% precast polyacrylamide gels (BioRad) before being transferred to a nitrocellulose membrane according to the manufacturer’s instructions. Loading was controlled by Ponceau’s staining before immunoblotting was performed by addition of the appropriate primary antibody, either an anti-GFP (anti-mVenus) polyclonal antibody (MBL #598), a custom made anti-BacA polyclonal antibody, or an anti-GAPDH polyclonal antibody (Proteogenix, France). Goat anti-rabbit HRP (Pierce) was used as the secondary antibody. Transferred blots were visualized with SuperSignal West Pico substrate (ThermoFisher Scientific) using a Bio-Rad Chemidoc.

## Supporting information

S1 Fig Analysis of the domain structure of bactofilins in the *Caulobacteraceae* family, and proposed *A. biprosthecum* BacA mutations. **A)** Multiple sequence alignment from Fig 1A highlighting proline residues (highlighted in red) in the proline-rich N- and C-terminal domains flanking the central bactofilin domain (DUF583). The Pfam-predicted bactofilin domain is delineated in magenta and the corrected bactofilin domain in mauve. **B)** Left: Structural prediction of *A. biprosthecum* BacA (green) using AlphaFold, with the Pfam-delimited bactofilin domain (DUF583) in magenta. Side, top, and bottom views of the structures are presented. Right: The predicted structure of *A. biprosthecum* BacA superimposed with the NMR-resolved structure of *C. crescentus* BacA (orange) (PDB-ID: 2N3D). **C)** High-resolution EM images of purified BacA and BacA_ΔNΔC_ filaments (scale bar = 1 µm). D) Phase contrast images of purified BacA_∆2–43;∆141–181_ (BacA_ΔNΔC_) filaments and BacA_∆2–43;∆139–181_ aggregates (scale bar = 20 µm). E) Schematic of the full-length BacA protein from *A. biprosthecum* and the proposed mutants for this study, based on the corrected bactofilin domain (residues 44–140).
(TIF)

S2 Fig BacA monomer and tetramer structural predictions. BacA monomer (top) and tetramer (bottom) predicted by AlphaFold, used in Figs 1 and 4 respectively. Structures are aligned and shown from the same orientation. Structures are ranked according to the predicted template modeling (pTM) score and are colored according to the predicted local distance difference test (pLDDT) score. Confidence in the prediction of each complex is indicated by the predicted aligned error (PAE) scores, which indicate positional error in angstroms for a given pair of residues across both protein chains.
(TIF)

S3 Fig Western blot and microscopy analysis of BacA terminal domain mutants. **A)** Top: Western blots using anti-GFP antibodies in mVenus-tagged BacA terminal domain deletion strains in *A. biprosthecum*. Strain free labeling was used as a loading control. Bottom: Western blots using anti-BacA antibodies in untagged terminal domain deletion strains in *A. biprosthecum* showed that antibodies raised against full length BacA protein were unable to detect truncated BacA mutants. Anti-GAPDH antibodies were used as a loading control. **B)** Atomic force microscopy of *A. biprosthecum bacA-mVenus*, Δ*bacA*, *bacA*_Δ*N*_*-mVenus* and *bacA*_Δ*C*_*-mVenus* strains, used to analyze the width of the stalk base. Stalks/pseudostalks are indicated with red arrows. Cells were grown in phosphate-limited (HIGG) medium (see Methods). Scale bars = 1 μm. **C)** Bacterial two-hybrid (BACTH) assays. T25-SpmX or T25-BacA constructs were tested against SpmX or BacA fused to the T18 domain at either the N-terminus (left panels) or C-terminus (right panels). Upper panels show results at 26°C while lower panels show results at 30°C. Leucine zipper motif served as a positive control (+Ctrl, bottom right). Negative controls used unfused T25 or T18 domains
(TIF)

S4 Fig BacA termini mutant quantification and microscopy images. **A)** Merged phase contrast/fluorescence (left) and fluorescence (middle) microscopy images with localization heatmaps (right) of SpmX-mCherry in *A. biprosthecum* WT, *bacA*_*ΔN*_, and *bacA*_*ΔC*_ strains grown in 30 µM phosphate HIGG. The number of cells analyzed in each case is shown on the bottom left of each heatmap. Scale bars = 2 μm. **B)** Quantification of BacA-mVenus (top) and SpmX-mCherry (bottom) foci per cell in *bacA* termini mutants shown in Fig 3. Data represent three independent biological replicates. **C)** Phase contrast, (left) fluorescence (middle) and Merged phase contrast/fluorescence (right) microscopy images of *A. biprosthecum* WT BacA-mVenus or BacA_ΔC_-mVenus co-expressed with SpmX-mCherry. Scale bars = 2 μm
(TIF)

S5 Fig Validation of BacA polymerization point mutants through Western blot, microscopy, and AFM analysis. **A)** Western blot using anti-GFP antibodies in the BacA polymerization point mutants in *A. biprosthecum*. Cells lysates were loaded at the same level of protein. Anti-GAPDH is presented as a loading control. **B)** Phase-contrast, fluorescence, and merged microscopy images of BacA-mVenus and BacA I56R-mVenus, with LUTs matching those used for mutants displaying diffuse fluorescence presented in Fig 5A. **C)** High-resolution atomic force microscopy (AFM) images displaying purified BacA filaments alongside all polymerization mutants. Scale bar represents 1 µm. Height measurements range from 0-60 nm for all samples except the I56R mutant, which ranges from 0-500 nm.
(TIF)

S1 Table Strains used in this study. (PDF)

S2 Table Plasmids used in this study. (PDF)

S3 Table Raw data. Numeric data used for plots in Figs 1, 2, 4, 5 and S3.
(XLSX)

## References

[1] Wagstaff J, Löwe J. Prokaryotic cytoskeletons: protein filaments organizing small cells. Nat Rev Microbiol. 2018;16(4):187–201. doi: 10.1038/nrmicro.2017.153 29355854

[2] Amos LA, Löwe J. Overview of the Diverse Roles of Bacterial and Archaeal Cytoskeletons. Subcell Biochem. 2017;84:1–26. doi: 10.1007/978-3-319-53047-5_1 28500521

[3] Errington J. Bacterial morphogenesis and the enigmatic MreB helix. Nat Rev Microbiol. 2015;13(4):241–8. doi: 10.1038/nrmicro3398 25578957

[4] Margolin W. FtsZ and the division of prokaryotic cells and organelles. Nat Rev Mol Cell Biol. 2005;6(11):862–71. doi: 10.1038/nrm1745 16227976 PMC4757588

[5] Ausmees N, Kuhn JR, Jacobs-Wagner C. The bacterial cytoskeleton: an intermediate filament-like function in cell shape. Cell. 2003;115(6):705–13. doi: 10.1016/s0092-8674(03)00935-8 14675535

[6] Ramos-León F, Ramamurthi KS. Cytoskeletal proteins: lessons learned from bacteria. Phys Biol. 2022;19(2):10.1088/1478-3975/ac4ef0. doi: 10.1088/1478-3975/ac4ef0 35081523

[7] Curtis Z, Escudeiro P, Mallon J, Leland O, Rados T, Dodge A, et al. Halofilins as emerging bactofilin families of archaeal cell shape plasticity orchestrators. Proc Natl Acad Sci U S A. 2024;121(40):e2401583121. doi: 10.1073/pnas.2401583121 39320913 PMC11459167

[8] Deng X, Gonzalez Llamazares A, Wagstaff JM, Hale VL, Cannone G, McLaughlin SH, et al. The structure of bactofilin filaments reveals their mode of membrane binding and lack of polarity. Nat Microbiol. 2019;4(12):2357–68. doi: 10.1038/s41564-019-0544-0 31501539 PMC6881188

[9] Kühn J, Briegel A, Mörschel E, Kahnt J, Leser K, Wick S, et al. Bactofilins, a ubiquitous class of cytoskeletal proteins mediating polar localization of a cell wall synthase in Caulobacter crescentus. EMBO J. 2010;29(2):327–39. doi: 10.1038/emboj.2009.358 19959992 PMC2824468

[10] Lin L, Thanbichler M. Nucleotide-independent cytoskeletal scaffolds in bacteria. Cytoskeleton (Hoboken). 2013;70(8):409–23. doi: 10.1002/cm.21126 23852773

[11] Bulyha I, Lindow S, Lin L, Bolte K, Wuichet K, Kahnt J, et al. Two small GTPases act in concert with the bactofilin cytoskeleton to regulate dynamic bacterial cell polarity. Dev Cell. 2013;25(2):119–31. doi: 10.1016/j.devcel.2013.02.017 23583757

[12] Vasa S, Lin L, Shi C, Habenstein B, Riedel D, Kühn J, et al. β-Helical architecture of cytoskeletal bactofilin filaments revealed by solid-state NMR. Proc Natl Acad Sci U S A. 2015;112(2):E127-36. doi: 10.1073/pnas.1418450112 25550503 PMC4299214

[13] Shi C, Fricke P, Lin L, Chevelkov V, Wegstroth M, Giller K, et al. Atomic-resolution structure of cytoskeletal bactofilin by solid-state NMR. Sci Adv. 2015;1(11):e1501087. doi: 10.1126/sciadv.1501087 26665178 PMC4672760

[14] Zuckerman DM, Boucher LE, Xie K, Engelhardt H, Bosch J, Hoiczyk E. The bactofilin cytoskeleton protein BacM of Myxococcus xanthus forms an extended β-sheet structure likely mediated by hydrophobic interactions. PLoS One. 2015;10(3):e0121074. doi: 10.1371/journal.pone.0121074 25803609 PMC4372379

[15] Sycuro LK, Pincus Z, Gutierrez KD, Biboy J, Stern CA, Vollmer W. Peptidoglycan crosslinking relaxation promotes Helicobacter pylori’s helical shape and stomach colonization. Cell. 2010;141(5):822–33. doi: 10.1016/j.cell.2010.04.002 20510929 PMC2920535

[16] Hay NA, Tipper DJ, Gygi D, Hughes C. A novel membrane protein influencing cell shape and multicellular swarming of Proteus mirabilis. J Bacteriol. 1999;181(7):2008–16. 10094676 10.1128/jb.181.7.2008-2016.1999 PMC93611

[17] Jackson KM, Schwartz C, Wachter J, Rosa PA, Stewart PE. A widely conserved bacterial cytoskeletal component influences unique helical shape and motility of the spirochete Leptospira biflexa. Mol Microbiol. 2018;108(1):77–89. doi: 10.1111/mmi.13917 29363884 PMC5867249

[18] Koch MK, McHugh CA, Hoiczyk E. BacM, an N-terminally processed bactofilin of Myxococcus xanthus, is crucial for proper cell shape. Mol Microbiol. 2011;80(4):1031–51. doi: 10.1111/j.1365-2958.2011.07629.x 21414039 PMC3091990

[19] Lin L, Osorio Valeriano M, Harms A, Søgaard-Andersen L, Thanbichler M. Bactofilin-mediated organization of the ParABS chromosome segregation system in Myxococcus xanthus. Nat Commun. 2017;8(1):1817. doi: 10.1038/s41467-017-02015-z 29180656 PMC5703909

[20] Sichel SR, Bratton BP, Salama NR. Distinct regions of H. pylori’s bactofilin CcmA regulate protein-protein interactions to control helical cell shape. eLife. 2022;11:e80111. 10.7554/eLife.80111 PMC9507126 36073778

[21] Taylor JA, Sichel SR, Salama NR. Bent Bacteria: A Comparison of Cell Shape Mechanisms in Proteobacteria. Annu Rev Microbiol. 2019;73(73):457–80. 31206344 10.1146/annurev-micro-020518-115919

[22] Billini M, Biboy J, Kühn J, Vollmer W, Thanbichler M. A specialized MreB-dependent cell wall biosynthetic complex mediates the formation of stalk-specific peptidoglycan in Caulobacter crescentus. PLoS Genet. 2019;15(2):e1007897. doi: 10.1371/journal.pgen.1007897 30707707 PMC6373972

[23] Pöhl S, Osorio-Valeriano M, Cserti E, Harberding J, Hernandez-Tamayo R, Biboy J. A dynamic bactofilin cytoskeleton cooperates with an M23 endopeptidase to control bacterial morphogenesis. eLife. 2024;12:RP86577. 10.7554/eLife.86577 PMC10945521 38294932

[24] Cserti E, Rosskopf S, Chang Y-W, Eisheuer S, Selter L, Shi J, et al. Dynamics of the peptidoglycan biosynthetic machinery in the stalked budding bacterium Hyphomonas neptunium. Mol Microbiol. 2017;103(5):875–95. doi: 10.1111/mmi.13593 27997718

[25] Wagner JK, Setayeshgar S, Sharon LA, Reilly JP, Brun YV. A nutrient uptake role for bacterial cell envelope extensions. Proc Natl Acad Sci U S A. 2006;103(31):11772–7. doi: 10.1073/pnas.0602047103 16861302 PMC1544245

[26] Klein EA, Schlimpert S, Hughes V, Brun YV, Thanbichler M, Gitai Z. Physiological role of stalk lengthening in Caulobacter crescentus. Commun Integr Biol. 2013;6(4):e24561. doi: 10.4161/cib.24561 23986806 PMC3737751

[27] de Young KD, Stankeviciute G, Klein EA. Sugar-Phosphate Metabolism Regulates Stationary-Phase Entry and Stalk Elongation in Caulobacter crescentus. J Bacteriol. 2020;202(4):e00468-19. doi: 10.1128/JB.00468-19 31767777 PMC6989804

[28] Jiang C, Brown PJB, Ducret A, Brun YV. Sequential evolution of bacterial morphology by co-option of a developmental regulator. Nature. 2014;506(7489):489–93. doi: 10.1038/nature12900 24463524 PMC4035126

[29] Pate JL, Ordal EJ. The fine structure of two unusual stalked bacteria. J Cell Biol. 1965;27(1):133–50. doi: 10.1083/jcb.27.1.133 5857250 PMC2106800

[30] Poindexter JL, Cohen-Bazire G. The fine structure of stalked bacteria belonging to the family Caulobacteraceae. J Cell Biol. 1964;23(3):587–607. 14245437 10.1083/jcb.23.3.587 PMC2106549

[31] Wagner JK, Brun YV. Out on a limb: how the Caulobacter stalk can boost the study of bacterial cell shape. Mol Microbiol. 2007;64(1):28–33. 17376069 10.1111/j.1365-2958.2007.05633.x

[32] Caccamo PD, Jacq M, VanNieuwenhze MS, Brun YV. A division of labor in the recruitment and topological organization of a bacterial morphogenic complex. Curr Biol. 2020;30(20):3908-3922.e4. 10.1016/j.cub.2020.07.063 PMC7578058 32795444

[33] Kysela DT, Randich AM, Caccamo PD, Brun YV. Diversity Takes Shape: Understanding the Mechanistic and Adaptive Basis of Bacterial Morphology. PLoS Biol. 2016;14(10):e1002565. doi: 10.1371/journal.pbio.1002565 27695035 PMC5047622

[34] Whittenbury R, Dow CS. Morphogenesis and differentiation in Rhodomicrobium vannielii and other budding and prosthecate bacteria. Bacteriol Rev. 1977;41(3):754–808. doi: 10.1128/br.41.3.754-808.1977 334156 PMC414022

[35] Taylor JA, Bratton BP, Sichel SR, Blair KM, Jacobs HM, DeMeester KE, et al. Distinct cytoskeletal proteins define zones of enhanced cell wall synthesis in Helicobacter pylori. Elife. 2020;9:e52482. doi: 10.7554/eLife.52482 31916938 PMC7012605

[36] Yang DC, Blair KM, Taylor JA, Petersen TW, Sessler T, Tull CM, et al. A Genome-Wide Helicobacter pylori Morphology Screen Uncovers a Membrane-Spanning Helical Cell Shape Complex. J Bacteriol. 2019;201(14):e00724-18. doi: 10.1128/JB.00724-18 31036730 PMC6597387

[37] Lee J, Cox JV, Ouellette SP. The unique N-terminal domain of chlamydial bactofilin mediates its membrane localization and ring-forming properties. J Bacteriol. 2023;205(6):e00092-23. 10.1128/jb.00092-23 PMC10294636 37191556

[38] Liu Y, Karmakar R, Steinchen W, Mukherjee S, Bange G, Schäfer LV, et al. Membrane binding properties of the cytoskeletal protein bactofilin. eLife [Internet]. 2024 Nov 13 [cited 2025 Jul 14];13. Available from: https://elifesciences.org/reviewed-preprints/100749 10.7554/eLife.100749 PMC12448750 40970374

[39] Ramamurthi KS. Protein localization by recognition of membrane curvature. Curr Opin Microbiol. 2010;13(6):753–7. 20951078 10.1016/j.mib.2010.09.014 PMC2994992

[40] Ramamurthi KS, Losick R. Negative membrane curvature as a cue for subcellular localization of a bacterial protein. Proc Natl Acad Sci U S A. 2009;106(32):13541–5. doi: 10.1073/pnas.0906851106 19666580 PMC2726380

[41] Randich AM, Kysela DT, Morlot C, Brun YV. Origin of a core bacterial gene via co-option and detoxification of a phage lysin. Curr Biol. 2019;29(10):1634-1646.e6. doi: 10.1016/j.cub.2019.04.014 PMC6594375 31080080

[42] Poindexter JS. Selection for nonbuoyant morphological mutants of Caulobacter crescentus. J Bacteriol. 1978;135(3):1141–5. doi: 10.1128/jb.135.3.1141-1145.1978 690072 PMC222491

[43] Saltikov CW, Newman DK. Genetic identification of a respiratory arsenate reductase. Proc Natl Acad Sci U S A. 2003;100(19):10983–8. doi: 10.1073/pnas.1834303100 12939408 PMC196913

[44] Ducret A, Quardokus EM, Brun YV. MicrobeJ, a tool for high throughput bacterial cell detection and quantitative analysis. Nat Microbiol. 2016;1(7):16077. doi: 10.1038/nmicrobiol.2016.77 27572972 PMC5010025

[45] Chen IMA, Chu K, Palaniappan K, Pillay M, Ratner A, Huang J, et al. IMG/M v.5.0: an integrated data management and comparative analysis system for microbial genomes and microbiomes. Nucleic Acids Research. 2019;47(D1):D666-77. 10.1093/nar/gky901 PMC6323987 30289528

[46] Waterhouse AM, Procter JB, Martin DMA, Clamp M, Barton GJ. Jalview Version 2--a multiple sequence alignment editor and analysis workbench. Bioinformatics. 2009;25(9):1189–91. doi: 10.1093/bioinformatics/btp033 19151095 PMC2672624

[47] Hanson J, Paliwal KK, Litfin T, Zhou Y. SPOT-Disorder2: Improved Protein Intrinsic Disorder Prediction by Ensembled Deep Learning. Genomics Proteomics Bioinformatics. 2019;17(6):645–56. doi: 10.1016/j.gpb.2019.01.004 32173600 PMC7212484

[48] Erdős G, Pajkos M, Dosztányi Z. IUPred3: prediction of protein disorder enhanced with unambiguous experimental annotation and visualization of evolutionary conservation. Nucleic Acids Research. 2021;49(W1):W297-303. 10.1093/nar/gkab408 PMC8262696 34048569

[49] Kozlowski LP, Bujnicki JM. MetaDisorder: a meta-server for the prediction of intrinsic disorder in proteins. BMC Bioinformatics. 2012;13:111. doi: 10.1186/1471-2105-13-111 22624656 PMC3465245

[50] Walsh I, Martin AJM, Di Domenico T, Tosatto SCE. ESpritz: accurate and fast prediction of protein disorder. Bioinformatics. 2012;28(4). 10.1093/bioinformatics/btr682 22190692

[51] Mirdita M, Schütze K, Moriwaki Y, Heo L, Ovchinnikov S, Steinegger M. ColabFold: making protein folding accessible to all. Nat Methods. 2022;19(6):679–82. doi: 10.1038/s41592-022-01488-1 35637307 PMC9184281

[52] Pettersen EF, Goddard TD, Huang CC, Meng EC, Couch GS, Croll TI, et al. UCSF ChimeraX: Structure visualization for researchers, educators, and developers. Protein Sci. 2021;30(1):70–82. doi: 10.1002/pro.3943 32881101 PMC7737788

