# Supplementary figures and images for: Polymerization and flanking domains of the bactofilin BacA collectively regulate stalk formation in Asticcacaulis biprosthecum
Source: PLoS Genet. 2025 Aug 13;21(8):e1011542. doi: 10.1371/journal.pgen.1011542 (PMC12364344; doi:10.1371/journal.pgen.1011542)

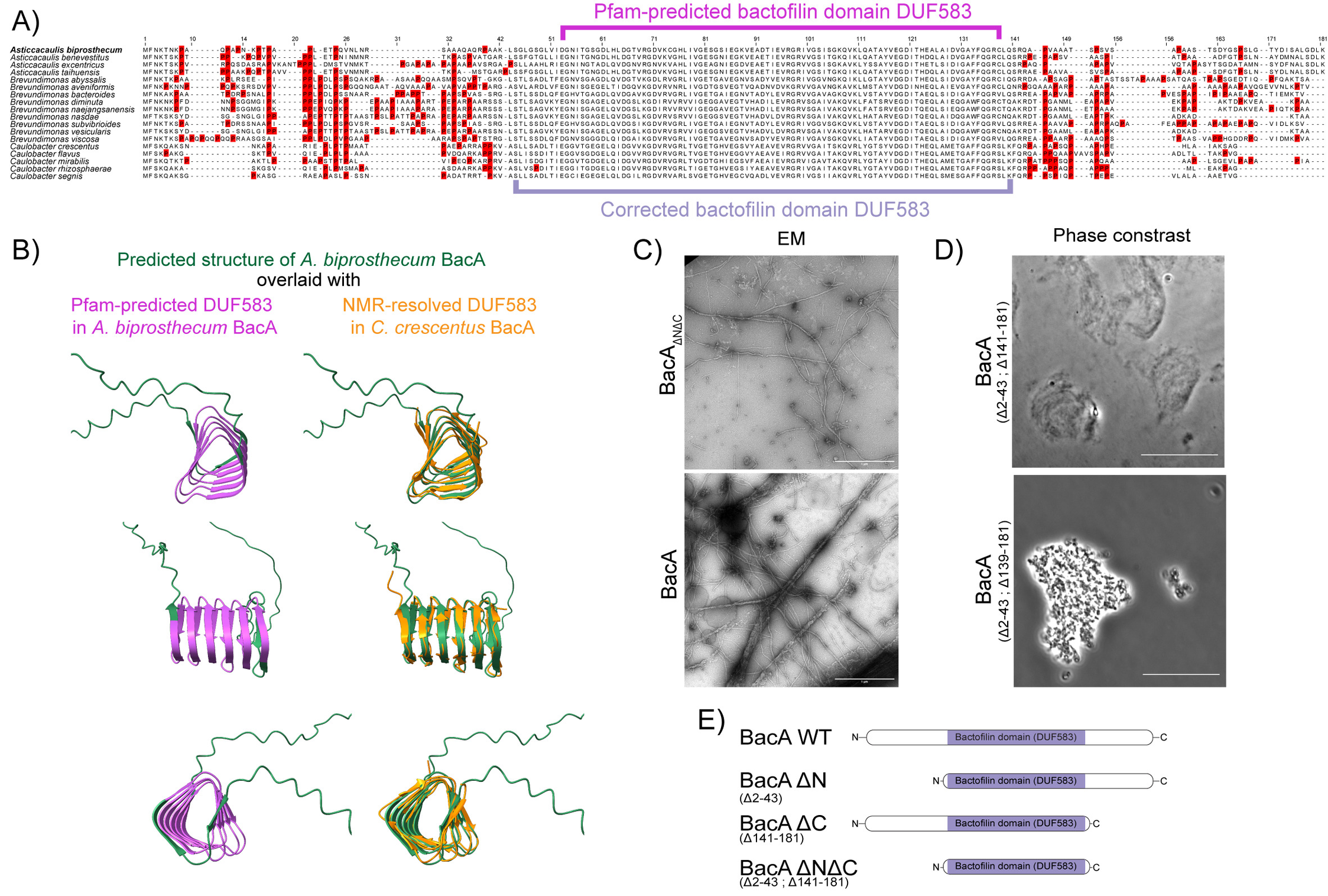

Supplement: S1 Fig — A) Multiple sequence alignment from Fig 1A highlighting proline residues (highlighted in red) in the proline-rich N- and C-terminal domains flanking the central bactofilin domain (DUF583). The Pfam-predicted bactofilin domain is delineated in magenta and the corrected bactofilin domain in mauve. B) Left: Structural prediction of A. biprosthecum BacA (green) using AlphaFold, with the Pfam-delimited bactofilin domain (DUF583) in magenta. Side, top, and bottom views of the structures are presented. Right: The predicted structure of A. biprosthecum BacA superimposed with the NMR-resolved structure of C. crescentus BacA (orange) (PDB-ID: 2N3D). C) High-resolution EM images of purified BacA and BacAΔNΔC filaments (scale bar = 1 µm). D) Phase contrast images of purified BacA∆2–43;∆141–181 (BacAΔNΔC) filaments and BacA∆2–43;∆139–181 aggregates (scale bar = 20 µm). E) Schematic of the full-length BacA protein from A. biprosthecum and the proposed mutants for this study, based on the corrected bactofilin domain (residues 44–140). (TIF) [file pgen.1011542.s001.tif]

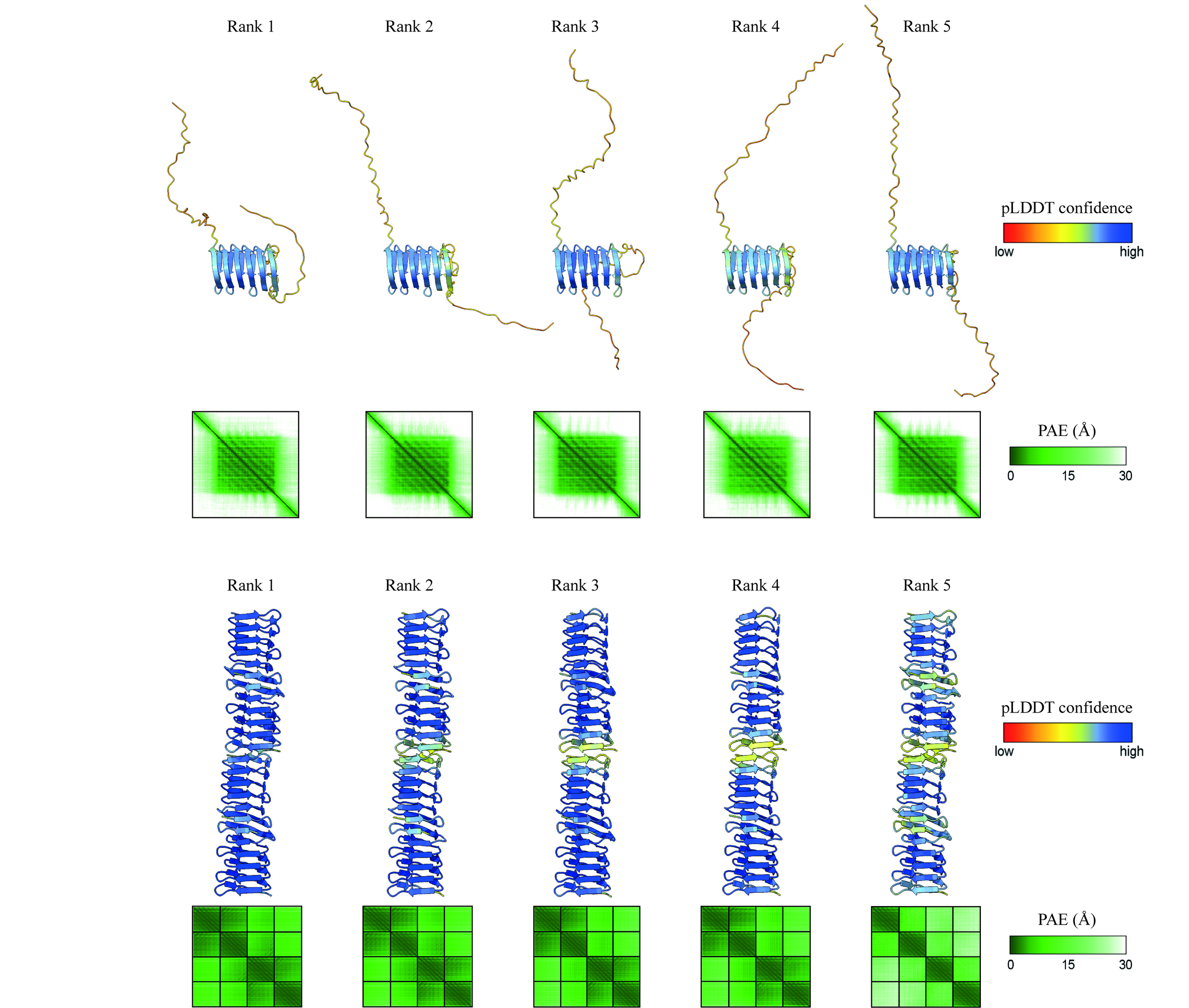

Supplement: S2 Fig — BacA monomer (top) and tetramer (bottom) predicted by AlphaFold, used in Figs 1 and 4 respectively. Structures are aligned and shown from the same orientation. Structures are ranked according to the predicted template modeling (pTM) score and are colored according to the predicted local distance difference test (pLDDT) score. Confidence in the prediction of each complex is indicated by the predicted aligned error (PAE) scores, which indicate positional error in angstroms for a given pair of residues across both protein chains. (TIF) [file pgen.1011542.s002.tif]

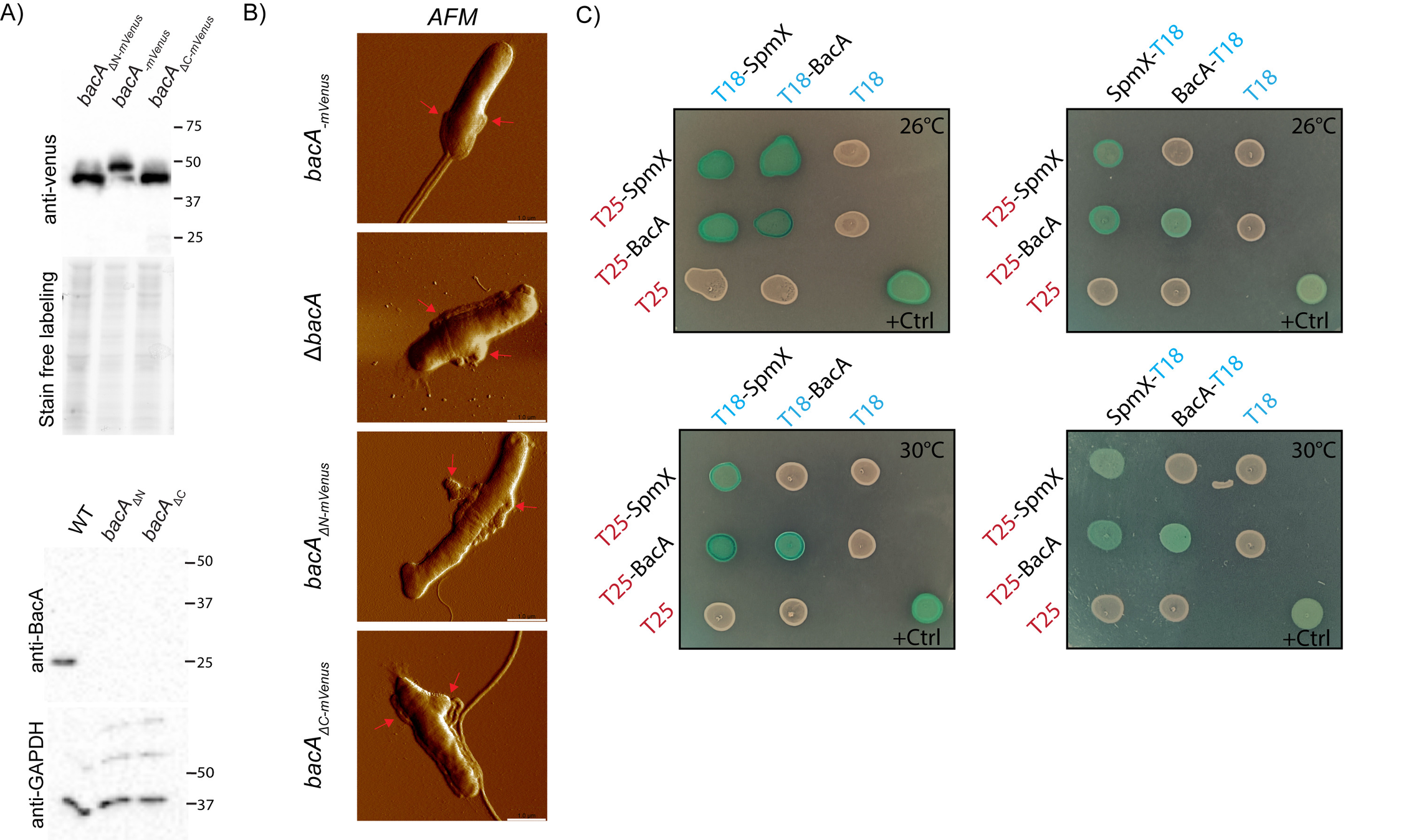

Supplement: S3 Fig — A) Top: Western blots using anti-GFP antibodies in mVenus-tagged BacA terminal domain deletion strains in A. biprosthecum. Strain free labeling was used as a loading control. Bottom: Western blots using anti-BacA antibodies in untagged terminal domain deletion strains in A. biprosthecum showed that antibodies raised against full length BacA protein were unable to detect truncated BacA mutants. Anti-GAPDH antibodies were used as a loading control. B) Atomic force microscopy of A. biprosthecum bacA-mVenus, ΔbacA, bacAΔN-mVenus and bacAΔC-mVenus strains, used to analyze the width of the stalk base. Stalks/pseudostalks are indicated with red arrows. Cells were grown in phosphate-limited (HIGG) medium (see Methods). Scale bars = 1 μm. C) Bacterial two-hybrid (BACTH) assays. T25-SpmX or T25-BacA constructs were tested against SpmX or BacA fused to the T18 domain at either the N-terminus (left panels) or C-terminus (right panels). Upper panels show results at 26°C while lower panels show results at 30°C. Leucine zipper motif served as a positive control (+Ctrl, bottom right). Negative controls used unfused T25 or T18 domains (TIF) [file pgen.1011542.s003.tif]

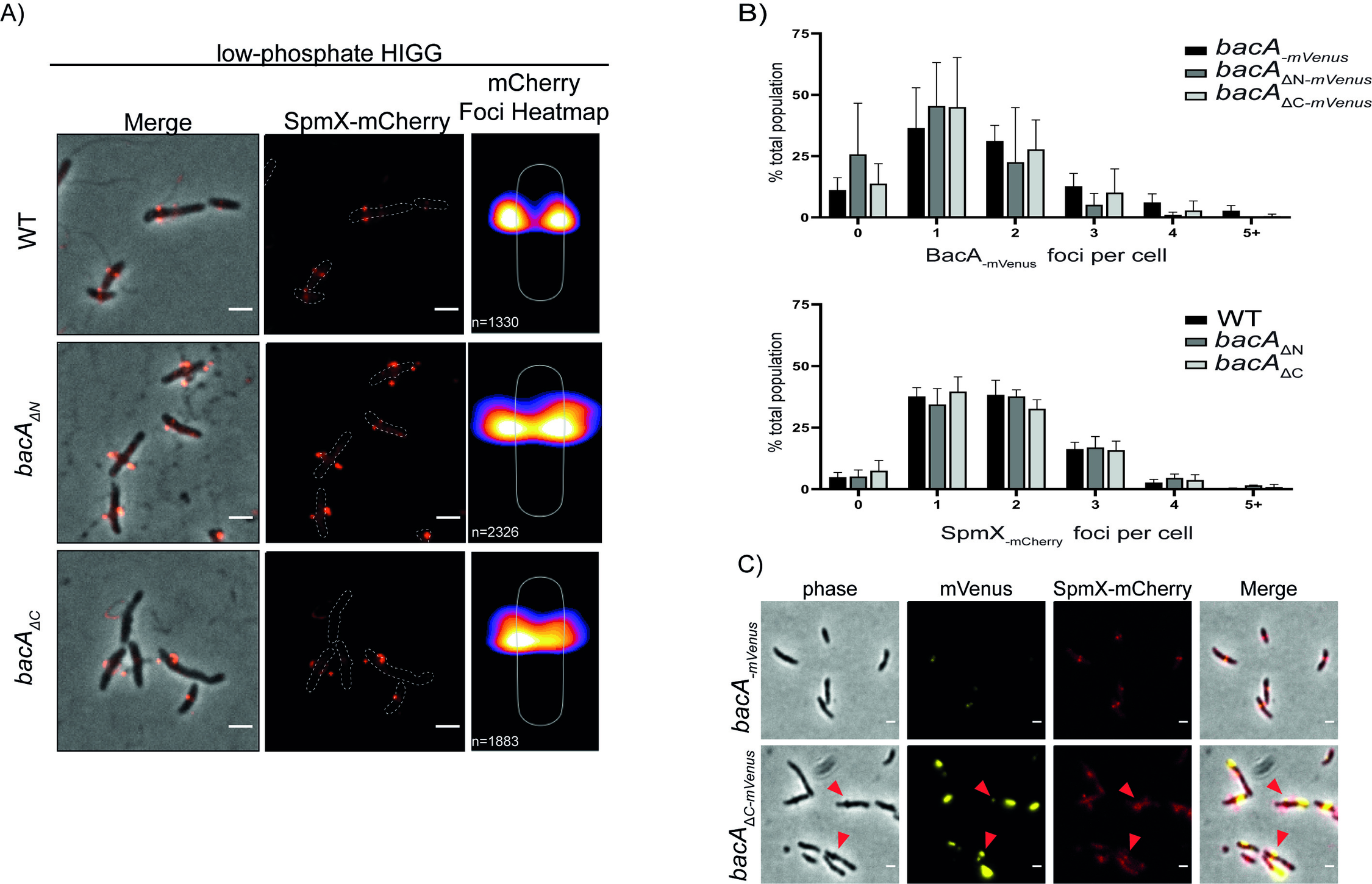

Supplement: S4 Fig — A) Merged phase contrast/fluorescence (left) and fluorescence (middle) microscopy images with localization heatmaps (right) of SpmX-mCherry in A. biprosthecum WT, bacAΔN, and bacAΔC strains grown in 30 µM phosphate HIGG. The number of cells analyzed in each case is shown on the bottom left of each heatmap. Scale bars = 2 μm. B) Quantification of BacA-mVenus (top) and SpmX-mCherry (bottom) foci per cell in bacA termini mutants shown in Fig 3. Data represent three independent biological replicates. C) Phase contrast, (left) fluorescence (middle) and Merged phase contrast/fluorescence (right) microscopy images of A. biprosthecum WT BacA-mVenus or BacAΔC-mVenus co-expressed with SpmX-mCherry. Scale bars = 2 μm (TIF) [file pgen.1011542.s004.tif]

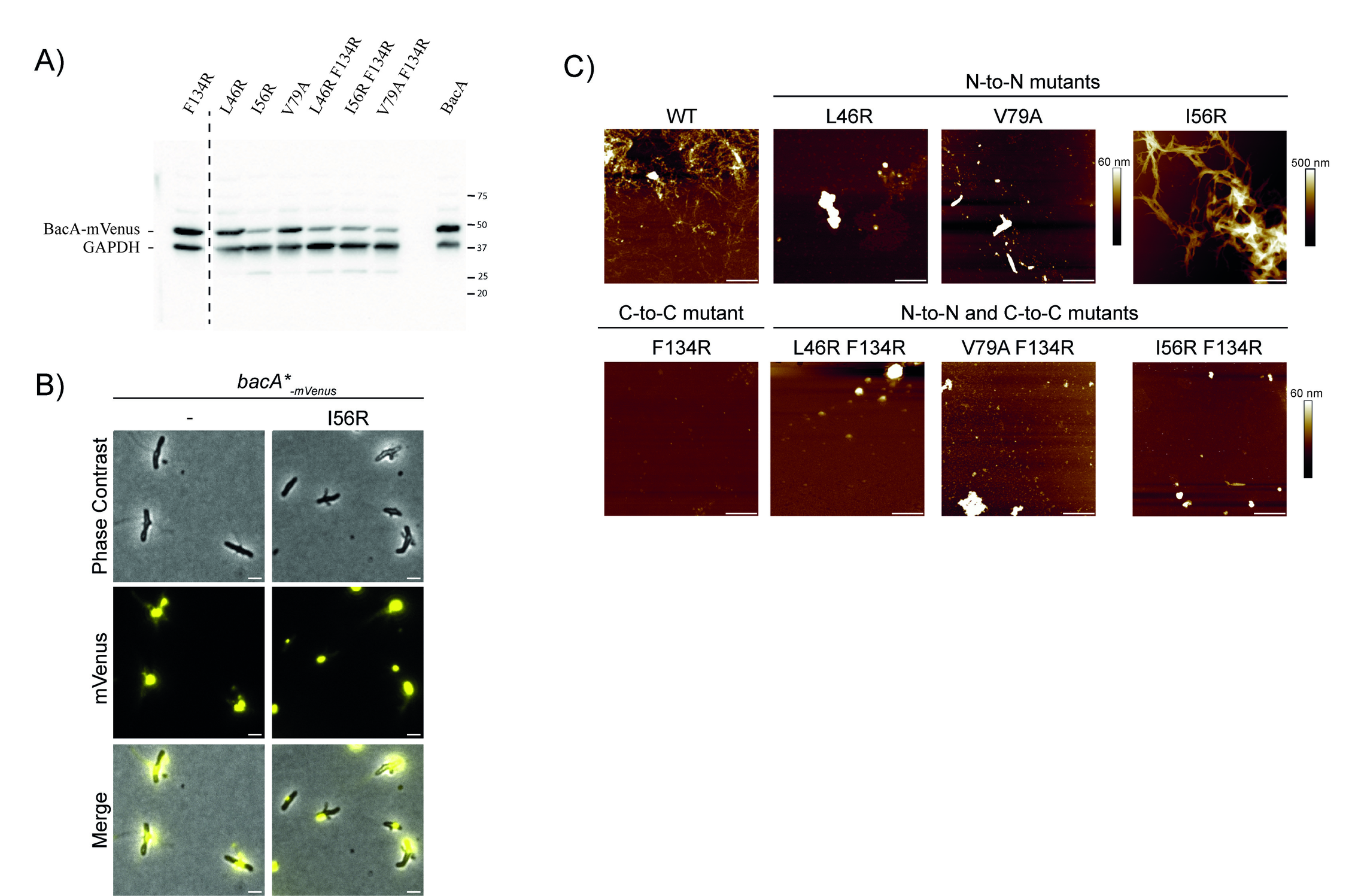

Supplement: S5 Fig — A) Western blot using anti-GFP antibodies in the BacA polymerization point mutants in A. biprosthecum. Cells lysates were loaded at the same level of protein. Anti-GAPDH is presented as a loading control. B) Phase-contrast, fluorescence, and merged microscopy images of BacA-mVenus and BacA I56R-mVenus, with LUTs matching those used for mutants displaying diffuse fluorescence presented in Fig 5A. C) High-resolution atomic force microscopy (AFM) images displaying purified BacA filaments alongside all polymerization mutants. Scale bar represents 1 µm. Height measurements range from 0-60 nm for all samples except the I56R mutant, which ranges from 0-500 nm. (TIF) [file pgen.1011542.s005.tif]
