# Supplementary material for: Polymerization and flanking domains of the bactofilin BacA collectively regulate stalk formation in Asticcacaulis biprosthecum
Source: PLoS Genet. 2025 Aug 13;21(8):e1011542. doi: 10.1371/journal.pgen.1011542 (PMC12364344; doi:10.1371/journal.pgen.1011542)
Supplement: S1 Table — (PDF) [file pgen.1011542.s006.pdf]

Table S1 – Strains used in this study.

Strain (YB#)	Genotype/description	Construction	Reference/source
E. coli			
**105**	Overexpression strain; BL21λDE3 (F- ompT hsdSB (rB-mB-) gal dcm (IDE3)		-
**7351**	DAP auxotroph; WM3064 (thrB1004 pro thi rpsL hsdS lacZΔM15 RP4-1360 Δ(araBAD)567 ΔdapA1341::[erm pir])		(43)
**9171**	BACTH strain; BTH101 (F-, cya-99, araD139, galE15, galK16, rpsL1 (Str r), hsdR2, mcrA1, mcrB1.)		Euromedex EUB001
A. biprosthecum			
**642**	WT C19		(29)
**8597**	Δ*bacA*	-	(32)
**9139**	*bacA (aa 45-181)-mVenus (ΔN)*	Created by electroporating and double recombination using pMJ77 into A. biprosthecum C19.	This study
**9141**	*bacA-mVenus*	Created by electroporating and double recombination using pMJ79 into A. biprosthecum C19.	This study
**9189**	*bacA(aa 1-140)-mVenus (ΔC)*	Created by electroporating and double recombination using pMJ76 into A. biprosthecum C19.	This study
**9506**	*bacA F134R-mVenus*	Created by electroporating and double recombination using pMJ125 into A. biprosthecum C19.	This study
**9507**	*bacA L46R-mVenus*	Created by electroporating and double recombination using pMJ124 into A. biprosthecum C19.	This study
**9510**	*bacA I56R-mVenus*	Created by electroporating and double recombination using pMJ123 into A. biprosthecum C19.	This study
**9512**	*bacA V79A mVenus*	Created by electroporating and double recombination using pMJ159 into A. biprosthecum C19.	This study
**9513**	*bacA V79A F134R-mVenus*	Created by electroporating and double recombination using pMJ156 into A. biprosthecum C19.	This study
**9594**	*bacA L46A F134R-mVenus*	Created by electroporating and double recombination using pMJ127 into A. biprosthecum C19.	This study
**9595**	*bacA I56A F134R-mVenus*	Created by electroporating and double recombination using pMJ128 into A. biprosthecum C19.	This study

**9895**	bacA (aa 1-140) (ΔC)	Created by electroporating and double recombination using pMJ169 into A. biprosthecum C19.	This study
**9896**	bacA (aa 45-181) (ΔN)	Created by electroporating and double recombination using pMJ170 into A. biprosthecum C19.	This study
**10188**	bacA (aa 1-140) (ΔC); spmX-mCherry	Created by electroporating and double recombination using pNPTS138-SpmX-mCherry into A. biprosthecum YB9895.	This study
**10189**	bacA (aa 45-181) (ΔN); spmX-mCherry	Created by electroporating and double recombination using pNPTS138-SpmX-mCherry into A. biprosthecum YB9896.	This study
**10190**	spmX-mCherry	Created by electroporating and double recombination using pNPTS138-SpmX-mCherry into A. biprosthecum C19.	This study
