# Supplementary material for: Polymerization and flanking domains of the bactofilin BacA collectively regulate stalk formation in Asticcacaulis biprosthecum
Source: PLoS Genet. 2025 Aug 13;21(8):e1011542. doi: 10.1371/journal.pgen.1011542 (PMC12364344; doi:10.1371/journal.pgen.1011542)
Supplement: S2 Table — (PDF) [file pgen.1011542.s007.pdf]

Table S2 – Plasmids used in this study

Plasmid	*E.coli* YB#	Genotype/description	Reference/source
**Overexpression plasmids**			
pET28a+		Vector carrying an N-terminal His•Tag/thrombin site for overexpression in *E.coli*	Novagen Cat#69864
pMJ4	8539	pET28a+ derivative containing His-tagged BacA (ABI_34180) in *E. coli*; Kan^R	(29)
pMJ26	9052	pET28a+ derivative containing His-tagged F134R in *E. coli*; Kan^R	This study
pMJ66	9107	pET28a+ derivative containing His-tagged BacA aa45-138 in *E. coli*; Kan^R	This study
pMJ71	9108	pET28a+ derivative containing His-tagged BacA aa45-140 in *E. coli*; Kan^R	This study
pMJ109	9140	pET28a+ derivative containing His-tagged I56R in *E. coli*; Kan^R	This study
pMJ110	9138	pET28a+ derivative containing His-tagged I56R F134R in *E. coli*; Kan^R	This study
pMJ119	9306	pET28a+ derivative containing His-tagged L46R in *E. coli*; Kan^R	This study
pMJ120	9311	pET28a+ derivative containing His-tagged L46R F134R in *E. coli*; Kan^R	This study
pMJ133	9522	pET28a+ derivative containing His-tagged V79A in *E. coli*; Kan^R	This study
pMJ157	9574	pET28a+ derivative containing His-tagged V79A F134R in *E. coli*; Kan^R	This study
**Bacterial two-hybrid plasmids**			
pKT25		Empty plasmid with MCS for C-terminal fusion with T25 fragment; Kan^R	Euromedex
pKNT25		Empty plasmid with MCS for N-terminal fusion with T25 fragment; Kan^R	Euromedex
pUT18		Empty plasmid with MCS for N-terminal fusion with T18 fragment; Amp^R	Euromedex
pUT18C		Empty plasmid with MCS for C-terminal fusion with T18 fragment; Amp^R	Euromedex
pKT25-zip		positive control with leucine zipper domain fused to T25 fragment; Kan^R	Euromedex
pUT-zip		Positive control with leucine zipper domain fused to T18 fragment; Amp^R	Euromedex
pPC62	9158	pKT25 derivative in which SpmX is fused to the T25 fragment; Kan^R	(29)
pPC63	9159	pKT25 derivative in which BacA is fused to the T25 fragment; Kan^R	(29)
pPC65	9162	pKNT25 derivative in which BacA is fused to the T25 fragment; Kan^R	(29)
pPC68	9165	pUT18 derivative in which BacA is fused to the T18 fragment; Amp^R	(29)

pPC72	9161	pKNT25 derivative in which SpmX is fused to the T25 fragment; Kan^R	(29)
pMJ105	9299	pKNT25 derivative in which BacA I56R is fused to the T25 fragment; Kan^R	This study
pMJ106	9300	pKNT25 derivative in which BacA I56R F134R is fused to the T25 fragment; Kan^R	This study
pMJ107	9301	pKT25 derivative in which BacA I56R is fused to the T25 fragment; Kan^R	This study
pMJ108	9302	pKT25 derivative in which BacA I56R F134R is fused to the T25 fragment; Kan^R	This study
pMJ111	9290	pUTC18 derivative in which BacA L46R F134R is fused to the T18 fragment; Amp^R	This study
pMJ114	9289	pUTC18 derivative in which BacA I56R is fused to the T18 fragment; Amp^R	This study
pMJ115	9307	pUTC18 derivative in which BacA I56R F134R is fused to the T18 fragment; Amp^R	This study
pMJ116	9288	pUT18 derivative in which BacA I56R is fused to the T18 fragment; Amp^R	This study
pMJ117	9314	pUTC18 derivative in which BacA I56R F134R is fused to the T18 fragment; Amp^R	This study
pMJ118	9315	pUTC18 derivative in which BacA L46R is fused to the T18 fragment; Amp^R	This study
pMJ139	9528	pKT25 derivative in which BacA L46R is fused to the T25 fragment; Kan^R	This study
pMJ140	9529	pKT25 derivative in which BacA L46R F134R is fused to the T25 fragment; Kan^R	This study
pMJ142	9559	pKNT25 derivative in which BacA L46R is fused to the T25 fragment; Kan^R	This study
pMJ143	9560	pKNT25 derivative in which BacA L46R F134R is fused to the T25 fragment; Kan^R	This study
pMJ146	9563	pUT18 derivative in which BacA L46R is fused to the T18 fragment; Amp^R	This study
pMJ147	9564	pUT18 derivative in which BacA L46R F134R is fused to the T18 fragment; Amp^R	This study
pMJ153	9570	pUT18C derivative in which BacA ΔN is fused to the T18 fragment; Amp^R	This study
pMJ154	9571	pUT18C derivative in which BacA ΔC is fused to the T18 fragment; Amp^R	This study
pMJ155	9572	pUT18C derivative in which BacA ΔNΔC is fused to the T18 fragment; Amp^R	This study
pMJ158	9575	pUT18C derivative in which BacA V79A is fused to the T18 fragment; Amp^R	This study
pMJ161	9578	pKT25 derivative in which BacA V79A is fused to the T25 fragment; Kan^R V79A	This study
pMJ162	9579	pUT18 derivative in which BacA V79A F134R is fused to the T18 fragment; Amp^R	This study
pMJ163	9580	pKNT25 derivative in which BacA V79A is fused to the T25 fragment; Kan^R	This study
pMJ164	9581	pKNT25 derivative in which BacA V79A F134R is fused to the T25 fragment; Kan^R	This study
pMJ165	9582	pUT18 derivative in which BacA V79A is fused to the T18 fragment; Amp^R	This study
pMJ167	9584	pKNT25 derivative in which BacA V79A F134R is fused to the T25 fragment; Kan^R	This study
pMJ168	9585	pUT18C derivative in which BacA V79A F134R is fused to the T18 fragment; Amp^R	This study

Integration or expression plasmid in *A. biprosthecum*

pMJ75	9124	pNPTS138 derivative used to generate bacA (aa 45-140)-mVenus (ΔN ΔC); Kan^R	This study
pMJ76	9125	pNPTS138 derivative used to generate bacA (aa 1-140)-mVenus (ΔC); Kan^R	This study
pMJ77	9126	pNPTS138 derivative used to generate bacA (aa 45-181)-mVenus (ΔN); Kan^R	This study
pMJ78	9127	pNPTS138 derivative used to generate bacA-mVenus (ATG differed); Kan^R	This study
pMJ79	9128	pNPTS138 derivative used to generate bacA-mVenus; Kan^R	This study
pMJ123	9500	pNPTS138 derivative used to generate bacA I56R -mVenus; Kan^R	This study
pMJ124	9501	pNPTS138 derivative used to generate bacA L46R -mVenus; Kan^R	This study
pMJ125	9502	pNPTS138 derivative used to generate bacA F134R -mVenus; Kan^R	This study
pMJ127	9504	pNPTS138 derivative used to generate bacA L46R F134R -mVenus; Kan^R	This study
pMJ128	9505	pNPTS138 derivative used to generate bacA I56R F134R -mVenus; Kan^R	This study
pMJ156	9573	pNPTS138 derivative used to generate bacA V79A F134R -mVenus; Kan^R	This study
pMJ159	9576	pNPTS138 derivative used to generate bacA V79A -mVenus; Kan^R	This study
pMJ169	9586	pNPTS138 derivative used to generate bacA (aa 1-140)(ΔC); Kan^R	This study
pMJ170	9587	pNPTS138 derivative used to generate bacA (aa 45-181) (ΔN); Kan^R	This study
pNPTS138-SpmX-mCherry		pNPTS138 derivative used to generate SpmX-mCherry; Kan^R	Vaidehi Patel
pCHYC-1-spmX_{AB}		pCHYC-1 bearing the C-terminal fragment of spmX (ABI_31540); Spec/StrepR	(25)
pNPTS138	1621	pLitmus derivative carrying *oriT* and *sacB*	M.R.K. Alley
